# Sequence-Specific Capture of Protein-DNA Complexes for Mass Spectrometric Protein Identification

**DOI:** 10.1371/journal.pone.0026217

**Published:** 2011-10-20

**Authors:** Cheng-Hsien Wu, Siyuan Chen, Michael R. Shortreed, Gloria M. Kreitinger, Yuan Yuan, Brian L. Frey, Yi Zhang, Shama Mirza, Lisa A. Cirillo, Michael Olivier, Lloyd M. Smith

**Affiliations:** 1 Department of Chemistry, University of Wisconsin, Madison, Wisconsin, United States of America; 2 Program in Cellular and Molecular Biology, University of Wisconsin-Madison, Madison, Wisconsin, United States of America; 3 Biotechnology and Bioengineering Center, Medical College of Wisconsin, Milwaukee, Wisconsin, United States of America; 4 Department of Biochemistry, Medical College of Wisconsin, Milwaukee, Wisconsin, United States of America; 5 Department of Cell Biology, Neurobiology, and Anatomy, Medical College of Wisconsin, Milwaukee, Wisconsin, United States of America; 6 Department of Physiology, Medical College of Wisconsin, Milwaukee, Wisconsin, United States of America; 7 Genome Center of Wisconsin, Madison, Wisconsin, United States of America; Instituto Nacional de Câncer, Brazil

## Abstract

The regulation of gene transcription is fundamental to the existence of complex multicellular organisms such as humans. Although it is widely recognized that much of gene regulation is controlled by gene-specific protein-DNA interactions, there presently exists little in the way of tools to identify proteins that interact with the genome at locations of interest. We have developed a novel strategy to address this problem, which we refer to as **GENECAPP**, for **G**lobal **E**xo**N**uclease-based **E**nrichment of **C**hromatin-**A**ssociated **P**roteins for **P**roteomics. In this approach, formaldehyde cross-linking is employed to covalently link DNA to its associated proteins; subsequent fragmentation of the DNA, followed by exonuclease digestion, produces a single-stranded region of the DNA that enables sequence-specific hybridization capture of the protein-DNA complex on a solid support. Mass spectrometric (MS) analysis of the captured proteins is then used for their identification and/or quantification. We show here the development and optimization of GENECAPP for an *in vitro* model system, comprised of the murine insulin-like growth factor-binding protein 1 (IGFBP1) promoter region and FoxO1, a member of the forkhead *rhabdomyosarcoma* (FoxO) subfamily of transcription factors, which binds specifically to the IGFBP1 promoter. This novel strategy provides a powerful tool for studies of protein-DNA and protein-protein interactions.

## Introduction

Proteins interact with DNA throughout the genome to control gene transcription on multiple levels (e.g. chromatin accessibility and recruitment of transcription machinery) [1]. Cis regulatory elements, that modulate the transcription of nearby genes, comprise only a fraction of the known regulatory sites controlled by transcription factors. Transcription of many genes is controlled by binding of proteins at distant sites, and a wide variety of protein-protein interactions within the chromatin provides additional levels of control by activating, enhancing or repressing transcription. Histone proteins can control chromatin accessibility and modulate secondary protein binding to DNA through various post-translational modifications. In spite of the critical importance of these protein-DNA interactions, at present few tools exist to identify and characterize the known and unknown proteins that interact with chromatin across the genome.

The most powerful technologies currently available employ chromatin immunoprecipitation, with subsequent analysis on DNA arrays (ChIP-Chip) [2], or by DNA sequencing (ChIP-Seq) [8], [9], [10] to identify DNA sequences that are directly or indirectly bound to proteins of interest throughout the genome. Although very effective and extremely useful, the greatest limitation of these strategies is their requirement for a specific antibody directed against the protein of interest. This limits the approach to characterizing the genome-binding behavior of already known proteins, and thus does not help to identify new, previously unknown proteins, nor does it help to reveal the identities of additional interacting proteins that are associated with particular genomic regions of interest.

New methods for mass-spectrometric identification of proteins binding to specific genomic loci are also beginning to emerge [11], [12], . Early attempts at accomplishing this involved exposure of synthetic dsDNA as bait to trap specific DNA-binding proteins from nuclear extract [13], [14], [16], [17]. The technique of SILAC (Stable Isotope Labeling by Amino acids in Cell culture) has been used to improve the confidence of such methods [15]. These *ex vivo* approaches have an advantage in that large amounts of DNA and extract can be used to isolate sufficient material for MS identification. In contrast, *in vivo* approaches are considerably more challenging because the DNA sequence of interest may be present at a level of as few as one copy per cell. Butala et al [11] were able to achieve successful identification of proteins from protein-DNA complexes formed *in vivo* in bacteria by increasing the abundance of the DNA through clever use of a low copy number plasmid containing the sequence of interest and LacI to facilitate extraction. Déjardin and Kingston [12] used locked nucleic acid (LNA) probes to isolate genomic DNA with its associated proteins. There, they captured telomeric sequences, which are highly repetitive regions at the end of chromosomes, to obtain sufficient material for protein identification. It remains to be seen if any of these methods can be multiplexed for parallel analysis of multiple gene sequences. Furthermore, none have yet demonstrated sensitivity for identification of *in vivo* bound DNA-binding proteins when the sequence of interest is present at only a single copy per cell.

We have developed a novel strategy to attack this problem, a strategy that is amenable to multiplexing and may offer single-copy sensitivity. We refer to it as **GENECAPP**, for **G**lobal **E**xo**N**uclease-based **E**nrichment of **C**hromatin-**A**ssociated **P**roteins for **P**roteomics. In this approach, formaldehyde cross-linking is employed to covalently link DNA to its associated proteins; subsequent fragmentation of the DNA, followed by exonuclease digestion, produces a single-stranded region of the DNA that enables sequence-specific hybridization capture of the protein-DNA complex on a solid support. Mass spectrometric (MS) analysis of the captured proteins is then used for their identification and/or quantification.

We describe here the development and optimization of this multi-step process for an *in vitro* model system, comprised of the murine insulin-like growth factor-binding protein 1 (IGFBP1) promoter region and FoxO1, a member of the forkhead *rhabdomyosarcoma* (FoxO) subfamily of transcription factors which binds specifically to IGFBP1. We prepared a 180 bp DNA PCR amplicon containing mouse IGFBP1 promoter sequence and formed a complex with recombinant FoxO1 protein prior to covalent cross-linking. This simulates the fragment that will be produced with fragmentation of native chromatin. Partial digestion of the FoxO1-IGFBP1 complex with exonuclease enables sequence-specific capture of the complex on a solid support grafted with complementary oligonucleotides. FoxO1 protein was subjected to protease digestion directly on the support, followed by MS analysis. This novel strategy provides a powerful tool for *in vitro* studies of DNA-protein and protein-protein interactions, and lays the foundation for further extensions of the approach for the comprehensive identification and quantitative analysis of proteins interacting with DNA *in vivo*.

## Results

### Global ExoNuclease-based Enrichment of Chromatin-Associated Proteins for Proteomics (GENECAPP)

**Figure 1:**
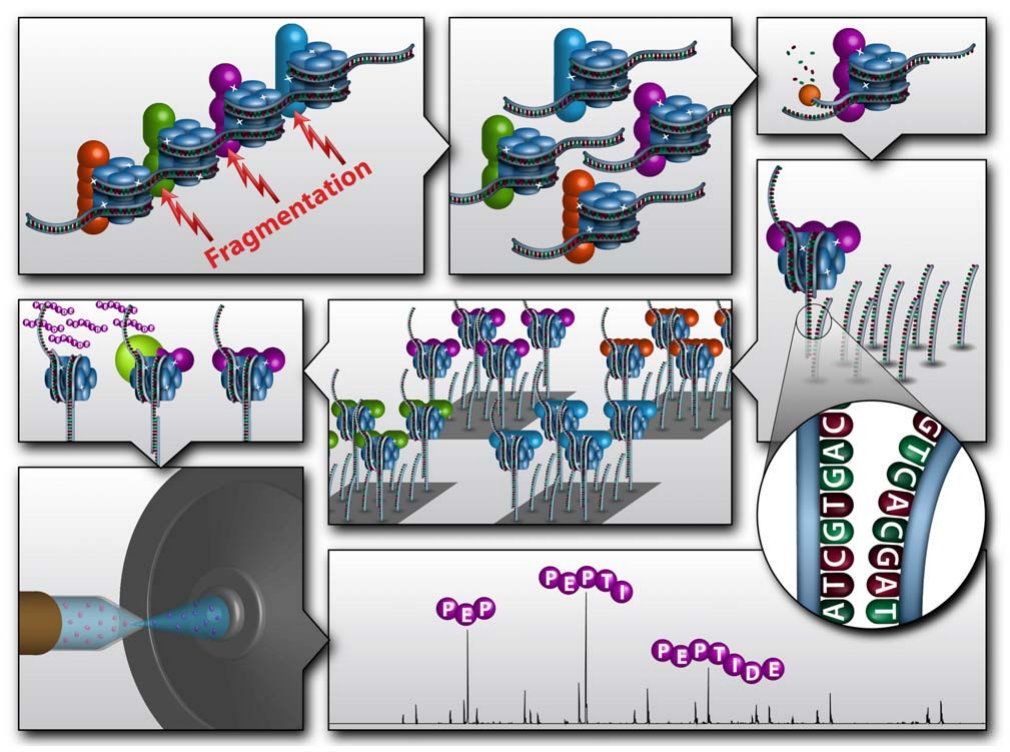
Schematic diagram of GENECAPP, for Global ExoNuclease-based Enrichment of Chromatin-Associated Proteins for Proteomics. In this illustration of the process, formaldehyde cross-linked chromatin is fragmented (e.g. sonication or restriction endonuclease) into small, nucleosome-length pieces. Fragments are treated with exonuclease to produce single-stranded regions, which are used for sequence-specific capture on a complementary DNA oligonucleotide array. Protease digestion of the captured complexes yields sample for MS analysis; enabling identification of the proteins and subsequent association of those proteins with genomic loci. Reproduced by permission from The Royal Society of Chemistry from Lloyd M. Smith, Michael R. Shortreed and Michael Olivier, *Analyst*, 2011, 136, 3060–3065, DOI: 10.1039/C1AN15037E.

**Figure 2:**
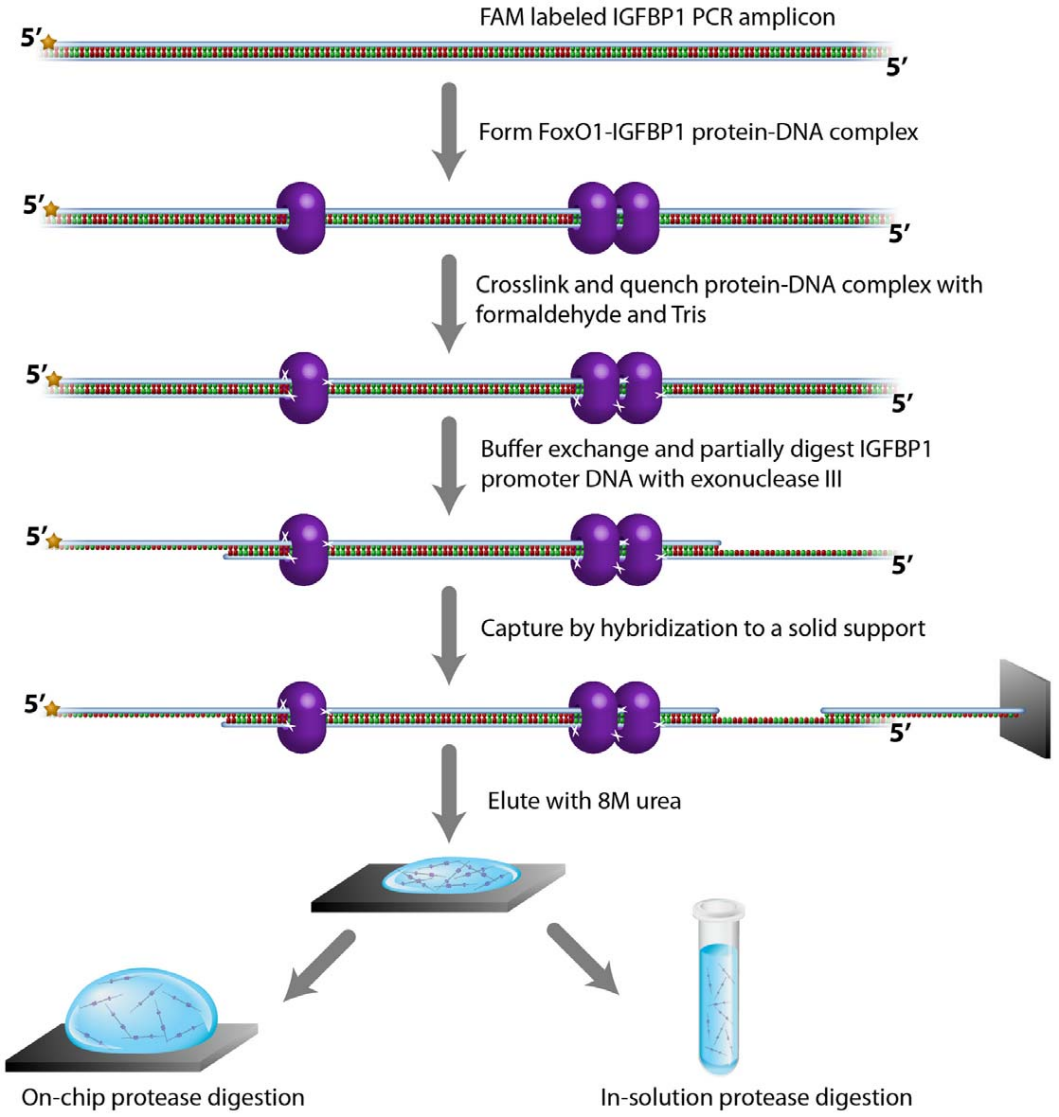
A model system for GENECAPP. FoxO1-IGFBP1 protein-DNA complex is formed in solution and cross-linked with formaldehyde prior to buffer exchange and exonuclease III digestion. The digestion produces 5′ single-stranded DNA overhangs for capture by hybridization on an *in situ* synthesized oligonucleotide array. The captured protein-DNA complexes are denatured using urea, protease digested either directly on the substrate or in solution, and then analyzed by MS. Initial experiments comparing on-chip versus in-solution digestion yielded little or no MS signal for the latter case; thus, we employed only on-chip digestion in all work reported herein.

The GENECAPP strategy employs sequence-specific hybridization capture of a specific DNA fragment to allow the isolation and subsequent characterization of all proteins bound to that region (Figure 1, reproduced by permission from The Royal Society of Chemistry from Lloyd M. Smith, Michael R. Shortreed and Michael Olivier, *Analyst*, 2011, **136**, 3060–3065, **DOI**: 10.1039/C1AN15037E) [18]. The first step in the process is the treatment of cells or tissue with formaldehyde to cross-link proteins to DNA, as is routinely done in ChIP-Chip/Seq assays. The chromatin is then fragmented, by either a physical means such as sonication, or an enzymatic means such as restriction enzyme digestion. Exonuclease digestion of one of the two strands of the duplexes protruding from the complex produces a free single-stranded region suitable for DNA hybridization. Incubation of this material with a solid support modified with complementary single-stranded DNA capture probes results in specific binding of the chromatin fragments of interest along with associated proteins. Subsequent characterization of these bound proteins by standard proteomic mass spectrometry techniques permits identification and/or quantification of proteins that are bound to the targeted DNA region, and potentially the characterization of posttranslational protein modifications. The approach is amenable to parallelization by using the multiplex capabilities of either array-based or bead-based platforms with multiple capture oligonucleotide probes that are complementary to targeted DNA regions of interest. Key steps in this process (cross-linking, exonuclease digestion, sequence-specific capture and MS protein identification) were developed and optimized using an *in vitro* model system (Figure 2) as described below.

### FoxO1-IGFBP1 model system

**Figure 3:**
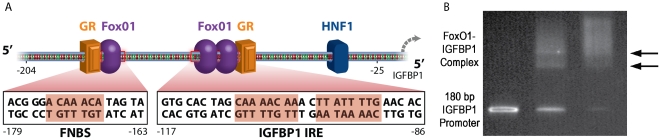
IGFBP1 promoter sequence and FoxO1 binding assay. (A) The 180 bp mouse IGFBP1 promoter sequence (−204 to −25) contains three FoxO1 binding sites, two within the IRE (insulin response element) and one new binding site designated FNBS. This promoter fragment also contains a binding site for the transcription factor HNF-1 (hepatocyte nuclear factor 1) and two binding sites for GR (glucocorticoid receptor). (B) Electrophoretic mobility shift assay (EMSA) confirms specific binding of recombinant FoxO1 protein to the PCR amplicon. The band in lane one corresponds to free IGFBP1 DNA. Two new bands, indicated by the arrows, appear in lane two when FoxO1 protein is mixed with the IGFBP1 DNA at a molar ratio of 1.5∶1.0 corresponding to the formation of the 1∶1 and 2∶1 FoxO1-IGFBP1 protein-DNA complex. Increasing the molar ratio of FoxO1 protein to IGFBP1 DNA to 3.0∶1.0 results in a nearly complete depletion of free DNA and an increase in the intensity of the band assigned to the 2∶1 complex.

FoxO1 protein binds to cognate sites within the insulin-like growth factor-binding protein 1 promoter [19], [20]. The sequence of the 180 bp PCR amplicon containing mouse IGFBP1 promoter sequences (−204 to −25) is shown in Figure 3A. This region of the IGFPB1 promoter contains an insulin response element (IRE), which has two FoxO1 cognate binding sites, and a third FoxO1 “new” binding site (FNBS) [20]. Specific binding of recombinant FoxO1 protein to the PCR amplicon was confirmed by electrophoretic mobility shift assay (EMSA) (Figure 3B). Two bands appear in the EMSA when FoxO1 protein is mixed with the IGFBP1 DNA at a molar ratio of 1.5∶1.0 (Figure 3B), corresponding to a 1∶1 FoxO1-DNA complex (lower band), and to a 2∶1 complex (upper band) [20]. Residual free DNA remains, consistent with the presence of multiple FoxO1 binding sites in the amplicon. Increasing the molar ratio of FoxO1 protein to IGFBP1 DNA to 3∶1 results in a nearly complete depletion of free DNA and an increase in the intensity of the band assigned to the 2∶1 complex. There is also increased intensity in the region of the gel above the second band suggesting that some of the DNA molecules have three or more proteins associated with them, although no distinct bands corresponding to such higher binding stoichiometries are observed. In control experiments using PCR amplicons lacking the FoxO1 cognate binding sites, no gel shift is observed (data not shown).

### Cross-linking proteins to DNA

Formaldehyde is widely used in ChIP, ChIP-Chip, and ChIP-Seq experiments to covalently cross-link protein-DNA, protein-protein and protein-RNA complexes and thereby maintain their integrity during processing [2], [3], [4], [5], [6], [7], [8], [9], [10], [21], [22], [23], [24], [25], [26], [27], [28], [29], [30], [31]. In order for it to have similar utility in GENECAPP, it is necessary that it not inhibit subsequent steps in the process, such as the exonuclease digestion of the DNA. To evaluate this we first determined the optimal concentration for formaldehyde cross-linking in the FoxO1-IGFBP1 model system. The FoxO1-IGFBP1 protein-DNA complex was prepared as described above and cross-linked for 10 min using various concentrations of formaldehyde (0.0–1.0% v/v), followed by quenching of the reaction with Tris (tris(hydroxymethyl)aminomethane) base (data not shown). The choice of Tris for quenching [32], as opposed to the more commonly used glycine [27], [28], [29], [30], [31], is discussed below. SDS (sodium dodecyl sulfate), an anionic surfactant, was added to a final concentration of 2.5% w/v followed by analysis on a tris-borate-EDTA (TBE) polyacrylamide gel. SDS is employed in order to denature any protein complexes that are not covalently linked by the formaldehyde treatment. At formaldehyde concentrations up to 0.625%, free dsDNA is present, whereas at concentrations of 0.75% or higher, no free DNA was observed, indicating complete cross-linking of the FoxO1-IGFBP1 complex. Accordingly, a concentration of 0.75% formaldehyde was employed for all subsequent cross-linking experiments.

### Quenching residual formaldehyde

**Figure 4:**
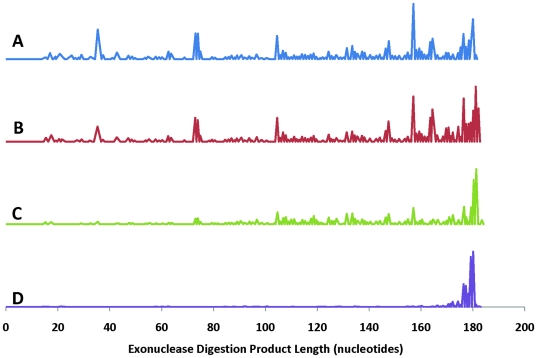
Fragment length analysis of exonuclease-digested DNA. The effect of cross-linking and quencher type was evaluated by profiling the exonuclease digestion products of FAM-labeled 180 bp IGFBP1 DNA: (A) untreated DNA control; (B) DNA cross-linked with 0.75% (v/v) formaldehyde; (C) DNA cross-linked with 0.75% (v/v) formaldehyde, followed by quenching with 250 mM Tris; and, (D) DNA cross-linked with 0.75% (v/v) formaldehyde, followed by quenching with 250 mM glycine.

Glycine is commonly used to quench formaldehyde cross-linking reactions in chromatin immunoprecipitation (ChIP) studies [27], [28], [29], [30], [31]. We found that DNA exposed to formaldehyde and then quenched with an equimolar amount of glycine, as is commonly done, becomes non-digestible by *E. coli* exonuclease III, which is a critical step in creating the single-stranded regions of DNA necessary for sequence-specific capture of the protein-DNA complex. 6-carboxyfluorescein (FAM)-labeled IGFBP1 dsDNA was treated with 0.75% formaldehyde at room temperature for 10 min. The solution was divided into three aliquots, one of which was kept as a non-quenched control, one was Tris-quenched, and the third was glycine-quenched. The buffer in all samples was exchanged for exonuclease III reaction buffer followed by exonuclease digestion. The digestion reaction was stopped after 0, 5 and 15 min by addition of EDTA, and the reaction products were both fragment length-analyzed (Figure 4, and Figures S1, S2, S3, S4) and characterized with respect to their hybridization to DNA tiling arrays (see below and Figures S5, S6, S7, S8). Exonuclease digestion of untreated DNA, formaldehyde treated DNA and formaldehyde-treated/Tris-quenched DNA produced very similar digestion product profiles on the gene sequencer. In contrast, the digestion product profile of the formaldehyde-treated and glycine-quenched DNA indicated a nearly complete absence of digestion.

### Exonuclease digestion of DNA and protein-DNA complexes

**Figure 5:**
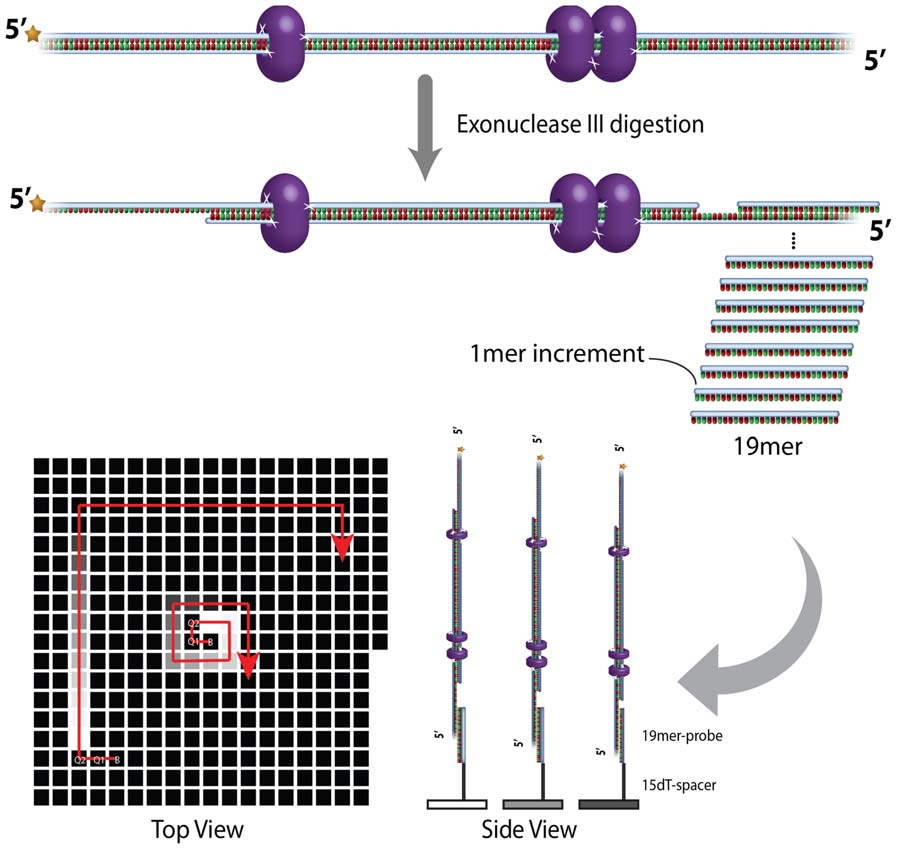
Schematic of tiling array design. An oligonucleotide tiling array containing all possible 19 mer complements, spanning the entire 180 bp long IGFBP1 DNA in 1 base steps, was fabricated using a maskless array synthesizer. The array was designed so that three quality control (non-complementary) sequences were placed directly in the center of the array. Probe one, which is complementary to the first 19 nt of the IGFBP1 DNA, was also located in the center of the array. The second probe, complementary to nucleotides 2–20, was placed directly to the right of probe one. Placement of additional probes proceeded in a clockwise spiral as shown by the red arrow. A second series of quality control and probe sequences were placed on the array beginning immediately after the first series was completed continuing in the clockwise spiral. FAM labeled protein-DNA complexes were pre-formed and partially digested by exonuclease III before being applied onto the tiling array for hybridization.

*E. coli* exonuclease III is a 3′ to 5′ exonuclease specific for double-stranded DNA [33], [34]. It is possible to obtain fine control over the digestion rate by controlling the enzyme dose, reaction time and temperature. We developed a DNA tiling array-based strategy (Figure 5) to characterize the products of the exonuclease digestion reaction. This allowed us to optimize the generation of single-stranded DNA for sequence-specific capture on the array. We fabricated an oligonucleotide tiling array containing all possible 19 mer complements, thereby spanning the entire 180 bp long IGFBP1 DNA in 162 single-base increments. The fluorescence signal that is observed necessarily arises from a partial duplex because the 5′-terminal FAM tag utilized for fluorescence detection is not on the DNA strand that directly hybridizes to the surface-bound capture probe. Fluorescence signal from each array element provides a measurement of the amount of digested duplex capturable by specific complementary oligonucleotides and was expected to vary with the degree of digestion. The integrated fluorescence signal summed from all features on the array provides a measure of the total amounts of capturable duplex allowing comparison between various treatment conditions. The digestion products were also analyzed by DNA fragment length analysis on a DNA sequencer (see Methods section), which provided quantitative information on the product length profile.

**Figure 6:**
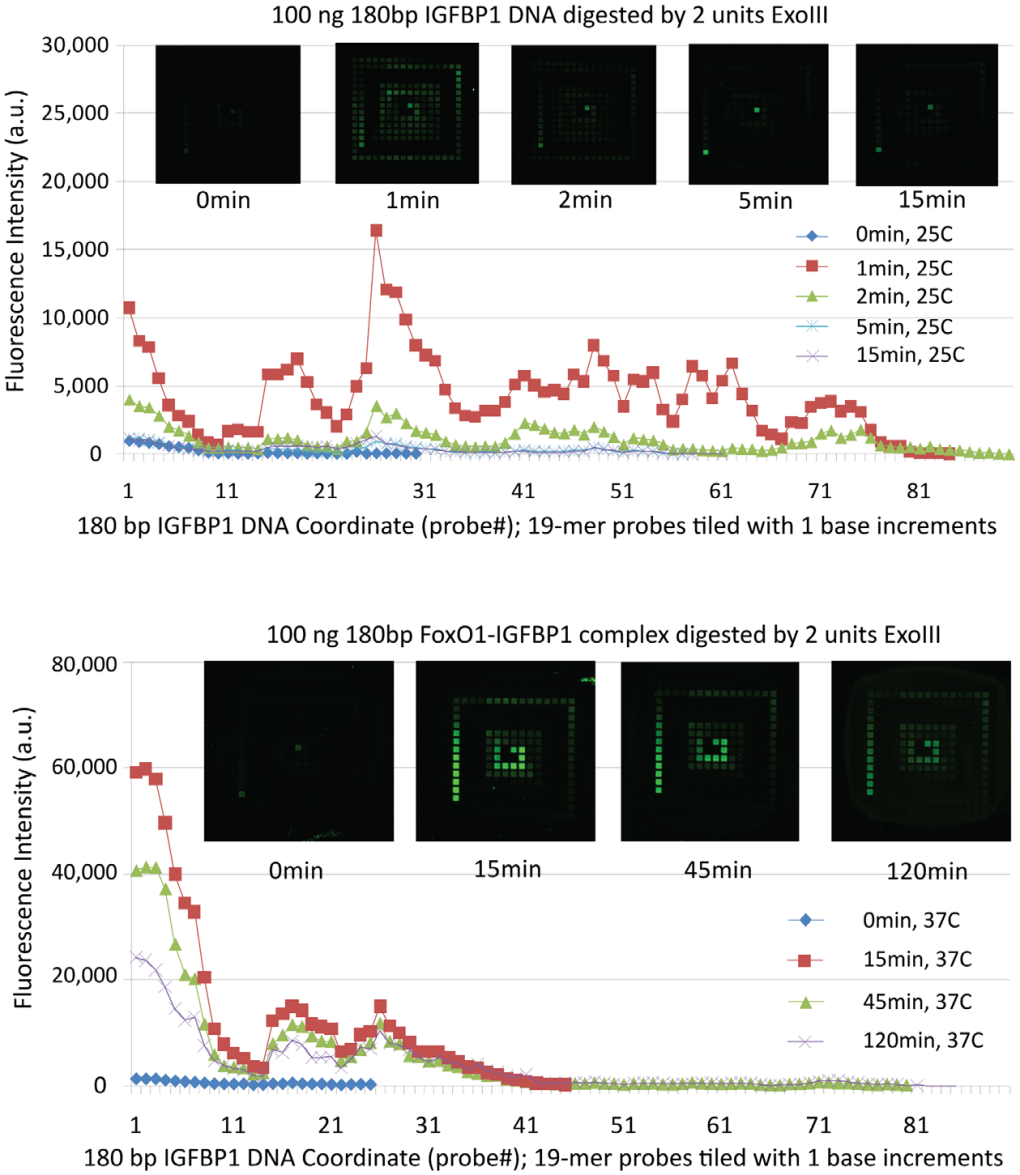
Tiling array profiles of exonuclease digested DNA. (A) The digestion profile of control FAM-labeled IGFBP1 DNA treated for 0, 1, 2, 5 and 15 min with exonuclease III at room temperature (25°C) is visualized by application of the product solution onto DNA tiling arrays and imaging the chip on a fluorescence scanner. The line profile directly below the tiling array images contains average intensities for the first 90 of 162 unique array features. Fluorescence signal from the remaining features was at background levels. (B) The digestion profile of the cross-linked and Tris-quenched FoxO1-FAM-labeled IGFBP1 DNA treated for 0, 15, 45 and 120 min with exonuclease III (37°C). Contrary to the digestion on free DNA, there is wide time window to digest cross-linked protein-DNA complex.

We profiled the digestion of two different IGFBP1 targets (pure IGFBP1 DNA and IGFBP1 DNA covalently cross-linked to FoxO1 protein with formaldehyde and quenched with Tris). Exonuclease III digestion of 100 ng pure IGFBP1 DNA by either 2 units of exonuclease III at room temperature for 1 min (Figure 6A) or by 0.2 units of exonuclease III at room temperature for 15 min (Figure S9) produced similarly large amounts of capturable duplex and relatively even digestion profiles (Figures 6, S1 and S9). Digestion of formaldehyde cross-linked and Tris-quenched FoxO1-IGFBP1 complex necessitated an increase in digestion time to between 15 and 45 minutes and an increase in reaction temperature to 37°C (Figure 6B). Higher or lower doses of exonuclease III generated lower amounts of capturable duplex presumably because of over- or under-digestion respectively. Similarly, longer reaction times resulted in over-digestion of both the pure duplex DNA (Figure S9) and the protein-DNA complex (Figure S10). The fragment length analysis data supported conclusions drawn from the tiling array analysis (Figures S11, S12, S13, S14, S15)

### Sequence-specific capture of FoxO1-IGFBP1 complex

The sequence-specific capture of exonuclease digested FoxO1-IGFBP1 complex was demonstrated on the DNA tiling arrays (Figure 6). Several non-complementary probe oligonucleotides were included on each array to monitor non-specific binding, which was found to be negligible. A major advantage of the tiling array is that the hybridization efficiency of many different capture probe sequences can be compared directly with one another, allowing identification of the best probe sequence for capture of the complex.

**Figure 7:**
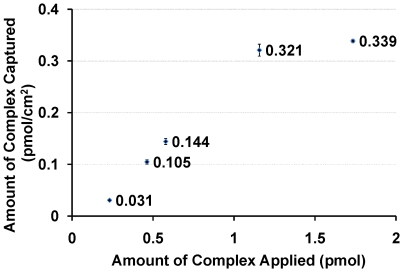
Capture of cross-linked FoxO1-IGFBP1 protein-DNA complex on a photolithographically-fabricated DNA array. Error bars correspond to the standard deviation of two replicate experiments.

The digested FoxO1-IGFBP1 complex bound most strongly to the end (first) oligonucleotide complement in the tiling array (Figure 6B). Further testing of the capture on array substrates covered entirely with a uniform layer of that complementary capture probe permitted a determination of the capacity of the surface to bind the protein-DNA complex (Figure 7). The oligonucleotide-modified substrate was treated sequentially with increasing concentrations of the FAM-labeled FoxO1-IGFBP1 complex. The surface was then rinsed briefly to remove any of the complexes that had not hybridized to the surface. An 8 M urea solution was used to denature the hybridization between the captured complex and the surface. This solution was collected and the amount of fluorescence was compared to fluorescence measured on a series of standard solutions prepared by mixing known amounts of the fluorescent complex with 8 M urea. The amount of complex captured compared with the amount applied is plotted in Figure 7. The amount of material captured levels off at about 0.33 pmol/cm^2^, and application of more than 1.5 pmol/cm^2^ yields no further increase in the amount captured. The capture efficiency can be calculated from the data in Figure 7 by dividing the amount of complex captured on the surface by the amount of complex that was applied. The maximum capture efficiency was slightly greater than 25% for the case where 1.5 pmol of complex was applied to the surface. The absolute amount of material captured was slightly larger when more complex was applied, but with a lower relative efficiency.

### Mass spectrometric protein identification and quantification

Captured FoxO1-IGFBP1 complex was digested with trypsin and analyzed by mass spectrometry. For discovery-based analysis (i.e. identification of unknown proteins), digests were analyzed on a linear ion trap/Orbitrap tandem mass-spectrometer operating in data-dependent acquisition (DDA) mode. For targeted, quantitative analysis, samples were analyzed on a triple-quadrupole/linear ion trap mass-spectrometer operating in SRM mode and using isotopically heavy labeled FoxO1 peptides spiked into samples to serve as internal standards. The signal from the respective heavy and light FoxO1 peptide SRM transitions were used to calculate the amount of protein in each sample. Both types of analysis yielded positive results, providing both robust identification (via decoy/target database searching with SEQUEST algorithm and filtration to a 1% false discovery rate) and quantification (see below) of FoxO1 protein.

**Figure 8:**
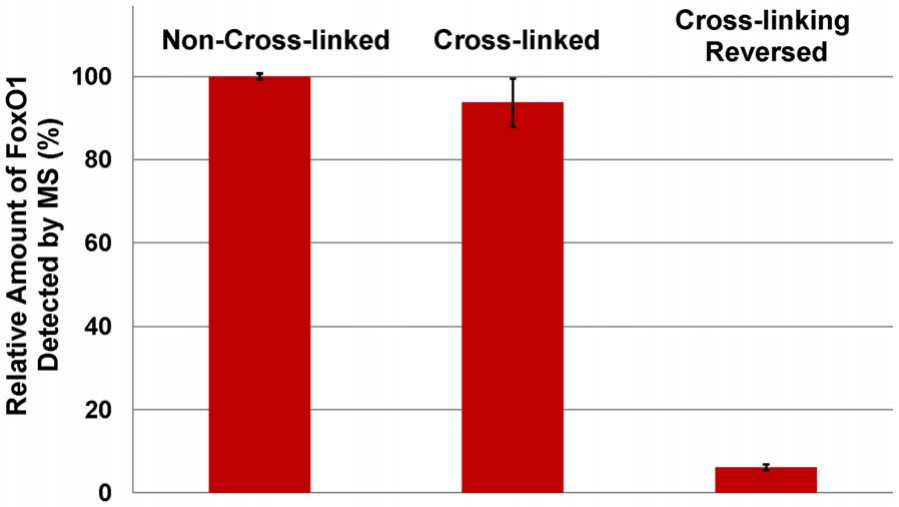
Normalized amount of FoxO1 protein detected by MS following cross-linking and cross-linking reversal. FoxO1-IGFBP1 protein-DNA complexes were prepared in solution at a 3∶1 molar ratio. One sample of the complex was cross-linked with formaldehyde. An aliquot of this sample was subsequently treated to reverse the cross-links (see Methods section). The MS signal obtained by SRM analysis of an untreated control sample was nearly equivalent to the MS signal from the cross-linked sample indicating that the cross-linking had only a limited effect. However, the MS signal obtained by SRM analysis of the cross-link reversed sample was considerably lower than either, indicating a loss of signal resulting from the cross-linking reversal procedure. Each sample was prepared in duplicate and then analyzed three separate times (technical replicates). Error bars in the graph represent one standard deviation calculated from those six results.

The effect of formaldehyde cross-linking and its reversal upon mass spectrometric identification or quantification of the FoxO1 protein was evaluated. A 3∶1 mixture of FoxO1 and IGFBP1 DNA was prepared and split into parts for three treatments: (1) untreated, (2) formaldehyde cross-linked, and (3) formaldehyde cross-linked followed by cross-linking reversal. The reverse cross-linking step was performed by heating at 99°C for 25 minutes in a reverse cross-link buffer [12] containing 250 mM Tris, pH 8.8, 0.5 M β-mercaptoethanol, and 1% *RapiGest*, an MS-compatible detergent substituting for SDS. The efficacy of these cross-linking reversal conditions was confirmed by DNA mobility analysis on a polyacrylamide gel (data not shown). Both SRM and DDA analysis showed little difference in the amount of FoxO1 protein detected between non-cross-linked and cross-linked samples (SRM data shown in Figure 8 and Tables S1, S2 and S3). This indicates that cross-linking has little or no adverse effect on the ability to quantify FoxO1 in the FoxO1-IGFBP1 complex. However, after the cross-linking reversal step, detected FoxO1 amounts were significantly reduced, suggesting that the conditions employed compromise protein detection, perhaps due to some combination of protein degradation, chemical modification, or precipitation (Figure 8). In view of these results, we decided to eliminate the cross-linking reversal step from the process, as it unnecessarily decreased protein signal, and provided no apparent benefit.

### MS analysis of FoxO1-IGFBP1 complex captured on a solid support

**Figure 9:**
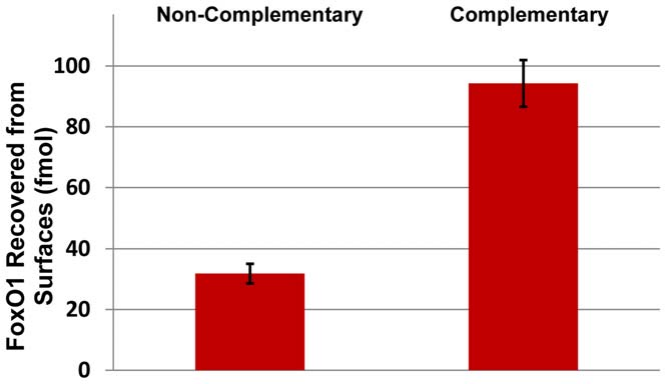
FoxO1 recovered from surfaces with complementary and non-complementary capture sequences.

We demonstrated the identification and quantification of FoxO1 protein from samples treated using the optimized GENECAPP process. FoxO1-DNA complexes were formed in solution, cross-linked with formaldehyde, quenched with Tris base, digested with exonuclease III and captured on a solid support grafted with complementary oligonucleotides. The surface-captured FoxO1-IGFBP1 complexes were digested on the solid support using an on-chip tryptic digestion procedure, which included incubation in concentrated urea to release the FoxO1-IGFBP1 complex from the surface and simultaneously denature FoxO1. Each sample was spiked with heavy labeled FoxO1 peptide standard and assayed by SRM, enabling accurate quantification of the amount of FoxO1 captured on the non-complementary versus complementary DNA-modified solid supports. As for the reverse-cross-linking experiments, each of these samples were also run on a linear ion trap/Orbitrap tandem mass-spectrometer in DDA mode and discovery results correlated well with SRM findings (data not shown). Substrates grafted with the complementary DNA probe oligonucleotide yielded approximately three-fold as much FoxO1 protein (94 fmol) as substrates with a non-complementary probe (32 fmol) (Figure 9). The FoxO1 levels in the FoxO1-IGFBP1 solution applied to each solid support were also determined and used to calculate capture recoveries of 16% and 5.4%, respectively.

## Discussion

We have developed and optimized a multistep process, GENECAPP, for the DNA sequence-based hybridization capture and mass spectrometric analysis of cross-linked protein-DNA complexes. Details of the process, as applied to an *in vitro* model protein-DNA complex, are discussed below.

### Cross-linking FoxO1 Protein to IGFBP1 DNA

A covalent bond between protein and DNA can preserve the specific binding relationship between the two molecules throughout several processing steps. For example, proteins are cross-linked to DNA in ChIP-Chip/Seq experiments prior to fragmentation of the chromatin. It is then possible to extract the relevant DNA fragments by immunoprecipitation of the target protein. Here, sequence-specific capture of the DNA is used to extract the desired complexes from solution, and cross-linking enables the associated proteins to remain attached to the DNA during the extraction and subsequent processing steps.

Formaldehyde offers several unique advantages as a cross-linker [21], [22], [28]. It rapidly permeates cell walls and membranes, covalently connects nucleophiles, weak and strong, which are ubiquitous in biological systems, and the cross-linking can be reversed if desired [21]. Formaldehyde is a reasonably specific cross-linking agent, in spite of its ability to form connections between essentially any nucleophile-containing molecules. This specificity arises from its very small size (∼2 Å), as only molecules that come into very close contact (e.g. specific protein-protein and protein-DNA complexes) can be cross-linked.

**Figure 10:**
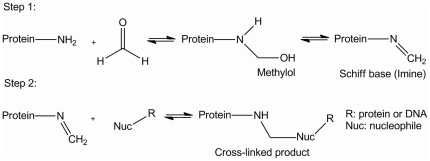
The general mechanism of formaldehyde cross-linking.

The general mechanism of formaldehyde cross-linking is shown in Figure 10. The first step is the reaction between formaldehyde and a relatively strong nucleophile, usually a primary amine, followed by dehydration of the methylol intermediate to yield an active Schiff-base. The second step of cross-linking is the reaction between this Schiff-base and another nucleophile, which can be a relatively weak one such as the amino group of a nucleic acid base. Through this two-step mechanism, formaldehyde is able to couple adjacent nucleophiles such as those found in the binding region of a protein-DNA complex.

FoxO1-IGFBP1 protein-DNA complexes were treated with increasing concentrations of formaldehyde during optimization of the cross-linking reaction and analyzed by polyacrylamide gel electrophoresis (data not shown). Low concentrations (below 0.375%) of formaldehyde were insufficient to covalently link all of the DNA duplexes in solution to protein molecules. At 0.375% formaldehyde the free DNA begins to disappear. We chose 0.75% formaldehyde as the optimum concentration, because it was the lowest concentration necessary for complete absence of free DNA. It is likely that the concentration of formaldehyde necessary for cross-linking *in vivo* will differ from the amount used here.

### Quenching residual formaldehyde

Glycine is commonly used to quench formaldehyde reactions in ChIP-Chip/Seq studies [7], [10]. It is thought that glycine stops cross-linking by reacting both with formaldehyde and with any active Schiff-base moieties (i.e. both Steps 1 and 2 in Figure 10). In the first case, glycine plus formaldehyde would form a Schiff-base, which then could react with another glycine. In the second case, Schiff-bases already formed on protein molecules could react with glycine, thereby preventing a cross-link between that protein and other protein or DNA molecules.

**Figure 11:**
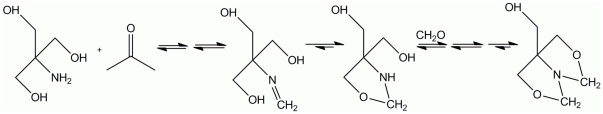
Quenching of formaldehyde cross-linking by Tris. The Schiff-base intermediate, formed from Tris's primary amine and formaldehyde, is attacked by the neighboring hydroxyl group to form a highly favored 5-membered ring. This sequence of reactions is repeated to form a second ring and produce the stable end product, 1-aza-3,7-dioxabicyclo[3.3.0]octane-5-methanol.

We found that DNA treated with formaldehyde and quenched with glycine was not digestible with exonuclease III (Figure 4 and Figures S1, S2, S3, S4, S5, S6, S7, S8). We speculate that glycine reacts with formaldehyde to form active Schiff-bases, which then react with the weak nucleophiles found on the DNA bases. This sequence of reactions decorates the DNA with glycine molecules and renders it unsuitable as a substrate for exonuclease III. Fortunately, an alternative reagent is Tris base [32], which quenches formaldehyde cross-linking while retaining the digestibility of the DNA. Glycine and Tris both contain a primary amine to react with formaldehyde, but Tris also has additional nucleophiles (hydroxyl groups) to react with the Schiff-bases (Figure 11). This highly favorable intramolecular reaction causes Schiff-bases formed from Tris to be consumed internally and thus not react to produce any modifications of DNA molecules. This hypothesis is consistent with our results showing that Tris quenching of formaldehyde cross-linking has a negligible effect on exonuclease III activity (Figure 4).

### Exonuclease digestion of dsDNA

Commercially available exonucleases, which could potentially be employed to generate single-stranded DNA for capture by hybridization, include lambda exonuclease, T7 exonuclease and *E. coli* exonuclease III. Lambda exonuclease, a highly processive 5′ exonuclease [35], [36], proved to be inconsistent and inefficient in our preliminary experiments (data not shown). T7 exonuclease has the ability to remove 5′ mononucleotides from duplex DNA, which would have removed the fluorescent tag from our model DNA and thus prevented us from visualizing it. Therefore, it was not used in these studies. *E. coli* exonuclease III is a 3′ to 5′ exonuclease specific for double-stranded DNA [33], [34]. It has been shown to have relatively low processivity and a uniform digestion rate [36], [37]. By controlling the enzyme dose, reaction time and temperature, we were able to obtain a high degree of control over the exonuclease III digestion rate (Figure 6), and thus it was selected for use.

The use of DNA tiling arrays provided an excellent means to profile the products of the exonuclease digestion (Figure 5). We profiled the digestion of two different IGFBP1 targets (pure IGFBP1 DNA and IGFBP1 DNA covalently cross-linked to FoxO1 protein with formaldehyde and quenched with Tris). We tested several different reaction temperatures, reaction times and enzyme dosages for the three different targets. Data for all tested conditions is included in the supporting information and two illustrative data sets are shown in Figure 6. Exonuclease III rapidly digests the pure IGFBP1 DNA at room temperature (Figure 6A). However, the cross-linked and Tris-quenched FoxO1-IGFBP1 complex is much more resistant to digestion. Increased digestion temperatures and times are required to produce the single-stranded DNA necessary for sequence-specific capture (Figure 6B). It is also notable that digestion ceased at approximately position 40 in the sequence (nucleotides 40–58), which is adjacent to one of the FoxO1 binding sites. This is consistent with blocking of the enzyme digestion by the bound protein, as is commonly observed in DNase footprinting studies [19], [20].

### Sequence-specific capture of protein-DNA complexes

Sequence-specific hybridization capture is a critical component of this work. There are many types of solid supports and oligonucleotide immobilization chemistries that can be employed for hybridization capture. In the present work, we employed DNA microarrays fabricated on glass using a maskless array synthesizer (MAS) [38]. A major advantage of the MAS is that it permits synthesis of as many as 786,432 different oligonucleotides on a 1×1.4 cm chip, providing a high degree of versatility and control. We used the MAS technology to synthesize both the tiling arrays used for analysis of exonuclease digestion and much simpler surfaces consisting of a single capture probe (or control probe) to maximize capture capacity.

The tiling array proved useful for identifying which of the many different 19 mer complementary oligonucleotides yielded the highest amount of binding to the partially single-stranded protein-DNA complex. There is an advantage to directly testing hybridization behavior in this manner because some of the behavior is not readily predictable. For example, the plots of fluorescence intensity from captured fluorescent complex clearly show some structure (Figure 6). There is a high intensity peak for DNA hybridization of the exonuclease-digested IGFBP1 DNA to the 26^th^ complementary probe (Figure 6A). We expected initially that the relative intensity of the hybridization signals to different surface-bound complements would correlate with the free energy of the duplex formed between the complement on the surface and the digested DNA. However, we found little correspondence between the two, indicating that other factors (as yet unidentified) play an important role in the binding process, and underscoring the need for empirical determinations to identify optimal sequences for hybridization capture.

### On-chip protease digestion and mass spectrometry analysis

As described in the Results section above, we found that the FoxO1-IGFBP complexes captured by sequence-specific hybridization on MAS chips could be digested by trypsin directly on the substrate surface, eliminating the need for removing the sample from the solid-support prior to protease digestion. Both identification and quantification of captured protein were demonstrated.

### Conclusion

We have described here a novel strategy for the **G**lobal **E**xo**N**uclease-based **E**nrichment of **C**hromatin-**A**ssociated **P**roteins for **P**roteomics, or GENECAPP. GENECAPP is a multi-step process for the mass spectrometric identification and quantification of DNA-associated proteins. In this proof-of-principle study we utilized the binding between FoxO1 and the IGFBP1 promoter region as a model system for technology development. The FoxO1-IGFBP1 complexes were formed *in vitro*, and cross-linked with formaldehyde. Tris was added to quench excess formaldehyde and active Schiff base intermediates. Exonuclease III was used to create single-stranded overhangs for capture by hybridization on a photolithographically-fabricated DNA array, followed by on-chip tryptic digestion. The tryptic peptides were analyzed by LC-MS/MS on both a linear ion trap/Orbitrap mass-spectrometer (for protein identification) and a triple quadrupole/linear ion trap mass spectrometer (for quantitative SRM analysis). Reaction conditions for each step were optimized, including preparation of the protein-DNA complex, formaldehyde cross-linking, quenching, exonuclease digestion, surface capture, protease digestion, and mass spectrometry analysis. The specific hybridization capture of FoxO1 protein was demonstrated and mass spectrometric protein identification and quantification were successfully performed.

## Materials and Methods

### FoxO1 protein and IGFBP1 promoter region complex preparation

FoxO1 protein was purified as a His_10_-tagged recombinant protein from *E. coli* as described previously [20]. The DNA fragment corresponding to positions −205 to −25 of the mouse IGFBP1 promoter was amplified by PCR from NIH 3T3 (mouse embryonic fibroblast cell line) genomic DNA (purchased from New England Biolabs, MA, USA; we used no cell lines in this work) using the primers (5′–TTA GCT CCT GTC CCA GTC CAT-3′ and 5′–TAT GAA GGG CTG GCT GTG C–3′). A 6-carboxyfluorescein (FAM) tagged oligonucleotide (5′–FAM-TTA GCT CCT GTC CCA GTC CAT-3′) was used to produce a 180 bp fluorescently tagged IGFBP1 promoter DNA amplicon (Text S1). All primers were custom synthesized by IDT (Integrated DNA Technologies, IA, USA). The amplicon was gel purified using the Promega Wizard SV Gel and PCR Clean-up System (Promega, WI, USA) prior to protein-DNA complex formation. Binding reactions of FoxO1 protein to IGFBP1 promoter DNA were performed in final buffer conditions of 10 mM Tris-HCl, pH 7.5, 1 mM MgCl_2_, 5 mM DTT, 40 mM KCl, 0.5% glycerol, 1 mg/mL BSA, and 1% Ficoll at room temperature for 1 h. A typical binding reaction was performed with 10 pmol DNA (∼1200 ng) and 30 pmol FoxO1 protein in 100 µL binding buffer. Formation of the complexes was verified by electrophoretic mobility shift assay (EMSA). SYBR Gold nucleic acid gel stain (Invitrogen, OR, USA) was used to stain DNA in Tris-borate EDTA polyacrylamide gels.

### Formaldehyde cross-linking and quenching

Freshly prepared FoxO1-IGFBP1 complex was cross-linked by formaldehyde (Pierce, IL, USA) for 10 min at 25°C. A 0.75% (v/v) final concentration of formaldehyde was used in all cross-linking reactions unless otherwise stated. Excess formaldehyde and active Schiff-base intermediates were quenched by addition of Tris, pH 8.0 to a final concentration of 250 mM followed by incubation at room temperature for 10 min. Glycine was tested as a quencher in a similar fashion.

### Formaldehyde cross-linking reversal

Formaldehyde cross-links were reversed by incubation at 99°C for 25 min in 250 mM Tris, pH 8.8, 0.5 M β-mercaptoethanol, and 1% *Rapi*Gest™ SF Surfactant (Waters, MA, USA). These cross-linking reversal conditions were adapted from a previous report [12], [21]. We used 1% *Rapi*Gest™ SF surfactant as a replacement for 2% sodium dodecyl sulfate so that the solution would be compatible with mass spectrometry.

### Buffer exchange and exonuclease digestion

The cross-linked and quenched solution of FoxO1-IGFBP1 complex was desalted three times using a cellulose-based 10,000 molecular weight cut-off Amicon Ultra-0.5 mL centrifugal filter (Millipore, MA, USA) prior to exonuclease III digestion. Recovery efficiencies were typically higher than 95% (data not shown). Note: the glycine-quenched protein-DNA complex was not compatible with purification using the Amicon Ultra-0.5 mL filter due to a large loss of sample in the filter (data not shown). The desalted DNA or protein-DNA complex was volume adjusted to ∼12 ng/µL in a final buffer of 10 mM Bis-Tris-propane-HCl, pH 7.0, 10 mM MgCl_2_ and 1 mM DTT. Two units of *E. coli* exonuclease III (NEB, MA, USA) were used to digest 100 ng IGFBP1 promoter DNA (180 bp) for 1 min at 25°C or FoxO1-IGFBP1 promoter protein-DNA complex for 45 min at 37°C unless otherwise stated. Exonuclease III digestions were stopped by addition of EDTA to a final concentration of 25 mM. Lambda exonuclease (NEB, MA, USA) and T7 exonuclease (T7 Gene 6 Exonuclease, Affymetrix, CA, USA) were used in DNA digestion comparison experiments. The extent of digestion was monitored on DNA tiling arrays or by fragment length analysis on a 3730xl DNA analyzer (Applied Biosystems, CA, USA).

### *In situ* photolithographic oligonucleotide array design and synthesis

Glass microscope slides (Plain Micro Slides, VWR, PA, USA) were cleaned with 1 M sodium hydroxide prior to silanization. The slides were then silanized for 4 h in 2% (v/v) N-(2-triethoxysilylpropyl)-4-hydroxy-butyramide (Gelest, Inc., Morrisville, PA, USA) in stock solution (0.1% acetic acid in 95% ethanol). After being rinsed by stirring in fresh stock solution for 15 min, the slides were transferred to a pre-heated (120°C) oven for 2 h, and cured under vacuum overnight. Light-directed photolithographic synthesis was performed on the silanized glass slides with a digital micromirror-based Maskless Array Synthesis (MAS) system connected to a ABI Expedite™ 8909 Nucleic Acid Synthesis System (Applied Biosystems, CA, USA) as described previously [38], [39], [40]. Table S4 contains the probe sequences synthesized on the surface. All the oligonucleotides were synthesized *in situ* in the 3′→5′ direction on the silanized glass. A 15-thymidine spacer, which has been shown to increase hybridization efficiency [41], [42], was included at the 3′ end of every oligonucleotide. Each of the tiling arrays was composed of 334 features, sized 130 µm×130 µm, and separated by 50 µm. The size of the capture and negative control arrays was 1.4 mm×1.0 mm.

### Capture and detection on oligonucleotide arrays

Exonuclease III digested FAM-tagged IGFBP1 promoter DNA and FoxO1-IGFBP1 complexes were supplemented with 10× SSPE buffer to give a final 1× SSPE concentration (10 mM NaH_2_PO_4_, 150 mM NaCl, 1 mM EDTA, pH 7.4) before application to the oligonucleotide arrays. The hybridization reaction was performed in a humid chamber at room temperature for 3 h. The surfaces were then rinsed and incubated in 1× SSPE buffer at 37°C for 15 min to remove nonspecifically bound DNA and protein-DNA complexes. Images of fluorescence were obtained using a GeneTac UC 4×4 microarray scanner (Genomic Solutions, MI, USA).

### Determination of surface binding capacity

A ten-fold molar excess of FoxO1 protein was added to FAM-tagged IGFBP1 promoter DNA to ensure the complete absence of free DNA. The complexes were formaldehyde cross-linked, Tris quenched and buffer exchanged with exonuclease III working buffer before digestion. The complexes were then exonuclease III digested based on a ratio of 2 units of exonuclease III for 100 ng FAM-tagged 180 bp IGFBP1 promoter DNA. Various amounts of the complexes were applied to the IGFBP1 capture tiling array for hybridization capture. Nonspecifically-bound complexes were removed by incubating the surface in 1× SSPE buffer at 37°C for 15 min. The captured complexes were then eluted by incubating the surface in 8 M urea at room temperature for 30 min. The capture capacity of the array was determined using a previously published wash-off method [43]. The fluorescence intensities of the solutions containing fluorescent complexes that were eluted from the surface with urea were compared to calibration solutions containing known amounts of the fluorescent complex (10^−11^ to 10^−8^ M) to determine the number of moles of complex recovered from the surface. The amount recovered from the surface divided by the surface area was reported as the binding capacity.

### On-chip protease digestion and analysis by mass spectrometry

On-chip protease digestion of captured FoxO1 protein was performed after thoroughly rinsing the array surfaces to remove any non-specifically bound complex. A 20 µL aliquot of 8 M urea in 50 mM ammonium bicarbonate buffer at pH 8.0 was applied to the array. The sample was incubated for 30 min at room temperature to elute the DNA from the surface and to denature the FoxO1 protein. The solution on the surface was then diluted ten-fold with 50 mM ammonium bicarbonate buffer to lower the urea concentration to <1 M. Sequence grade modified trypsin (Promega, WI, USA) was added at a final protease∶protein ratio of 1∶20 (w/w) based on the original FoxO1 input. The on-chip tryptic digestion was carried out in a humid chamber at 37°C overnight. For SRM analysis, two C-terminal heavy labeled FoxO1 proteotypic peptides, which were pre-selected (Table S1) and synthesized (Ulm, ThermoFisher Scientific, Germany), were spiked directly into the mixture on-chip in known amounts. Each sample was then purified using OMIX C18 pipette tips (Varian, CA, USA) before analysis by mass spectrometry. SRM samples were separated on a nanocapillary column using a nanoACQUITY UPLC system (Waters, MA, USA). All columns were prepared in-house and packed with MAGIC C18AQ stationary phase (Michrom Bioresources, CA, USA). The sample was nanosprayed into an AB SCIEX QTRAP 5500 triple quadrupole mass spectrometer (AB SCIEX, CA, USA), which monitored multiple heavy and light transitions per peptide pair for quantification. Samples were also analyzed on an LTQ Oribitrap Velos mass spectrometer (Thermo Scientific, FL, USA) in discovery mode and peptides were identified with Proteome Discoverer software. False discovery rate determinations were performed using the Proteome Discoverer software Decoy Database Search.

## Supporting Information

Figure S1 **Fragment length profile from digestion of dsDNA with exonuclease III as a function of time.** Two units of exonuclease III were used to digest 100 ng of FAM-labeled IGFBP1 DNA for 0, 5 and 15 min at room temperature. Exonuclease III digestions were stopped by addition of EDTA to a final concentration of 25 mM. The samples were subjected to fragment analysis using an ABI 3130xl Genetic Analyzer (Applied Biosystems, CA, USA).
(TIF)

Figure S2 **Fragment length profile from digestion of formaldehyde-treated dsDNA with exonuclease III as a function of time.** FAM-labeled 180 bp IGFBP1 DNA was pretreated with 0.75% (v/v) formaldehyde for 10 min. The excess formaldehyde was diluted and buffer exchanged before exonuclease III digestion. Two units of exonuclease III were used to digest the DNA for 0, 5 and 15 min at room temperature. After digestion, the samples were subjected to fragment analysis using an ABI 3130xl Genetic Analyzer (Applied Biosystems, CA, USA).
(TIF)

Figure S3 **Fragment length profile from digestion of formaldehyde-treated and Tris-quenched dsDNA with exonuclease III as a function of time.** The effect of cross-linking reagent (formaldehyde) and Tris quencher was evaluated by profiling the exonuclease digestion products. FAM-labeled 180 bp IGFBP1 DNA was pretreated with 0.75% (v/v) formaldehyde for 10 min, followed by quenching with 250 mM Tris. The formaldehyde and Tris were diluted and buffer exchanged. Two units of exonuclease III were used to digest the DNA for 0, 5 and 15 min at room temperature. After digestion, the samples were subjected to fragment analysis using an ABI 3130xl Genetic Analyzer (Applied Biosystems, CA, USA).
(TIF)

Figure S4 **Fragment length profile from digestion of formaldehyde-treated and glycine-quenched dsDNA with exonuclease III as a function of time.** The effect of cross-linking reagent (formaldehyde) and glycine quencher was evaluated by profiling the exonuclease digestion products. FAM-labeled 180 bp IGFBP1 DNA was pretreated with 0.75% (v/v) formaldehyde for 10 min, followed by quenching with 250 mM glycine. The formaldehyde and glycine were diluted and buffer exchanged. 2 units of exonuclease III were used to digest the DNA for 0, 5, and 15 min at room temperature. After digestion, the samples were subjected to fragment analysis using an ABI 3130xl Genetic Analyzer (Applied Biosystems, CA, USA).
(TIF)

Figure S5 **Fragment length profile from digestion of dsDNA with exonuclease III as a function of time.** Two units of exonuclease III were used to digest FAM-labeled IGFBP1 DNA for 0, 5 and 15 min at room temperature. The digestion profile was visualized by application of the product solution onto DNA tiling arrays and imaging the substrate on a fluorescence scanner. The line profile directly below the tiling array images contains average intensities for the first 90 of 162 unique array features. Fluorescence signal from the remaining features was at background levels.
(TIF)

Figure S6 **Fragment length profile from digestion of formaldehyde-treated dsDNA with exonuclease III as a function of time.** FAM-labeled 180 bp IGFBP1 DNA was pretreated with 0.75% (v/v) formaldehyde for 10 min. The excess formaldehyde was diluted and buffer exchanged before exonuclease III digestion. Two units of exonuclease III were used to digest the DNA for 0, 5 and 15 min at room temperature. The digestion profile was visualized by application of the product solution onto DNA tiling arrays and imaging the substrate on a fluorescence scanner. The line profile directly below the tiling array images contains average intensities for the first 90 of 162 unique array features. Fluorescence signal from the remaining features was at background levels.
(TIF)

Figure S7 **Fragment length profile from digestion of formaldehyde-treated and Tris-quenched dsDNA with exonuclease III as a function of time.** FAM-labeled 180 bp IGFBP1 DNA was pretreated with 0.75% (v/v) formaldehyde for 10 min and quenched with 250 mM Tris. The formaldehyde and Tris were diluted and buffer exchanged before exonuclease III digestion. Two units of exonuclease III were used to digest the DNA for 0, 5 and 15 min at room temperature. The digestion profile was visualized by application of the product solution onto DNA tiling arrays and imaging the substrate on a fluorescence scanner. The line profile directly below the tiling array images contains average intensities for the first 90 of 162 unique array features. Fluorescence signal from the remaining features was at background levels.
(TIF)

Figure S8 **Fragment length profile from digestion of formaldehyde-treated and glycine-quenched dsDNA with exonuclease III as a function of time.** FAM-labeled 180 bp IGFBP1 DNA was pretreated with 0.75% (v/v) formaldehyde for 10 min and quenched with 250 mM glycine. The formaldehyde and glycine were diluted and buffer exchanged before exonuclease III digestion. Two units of exonuclease III were used to digest the DNA for 0, 5 and 15 min at room temperature. The digestion profile was visualized by application of the product solution onto DNA tiling arrays and imaging the substrate on a fluorescence scanner. The line profile directly below the tiling array images contains average intensities for the first 90 of 162 unique array features. Fluorescence signal from the remaining features was at background levels.
(TIF)

Figure S9 **Fragment length profile from digestion of dsDNA with exonuclease III as a function of time and enzyme dosage.** The digestion profile was visualized by application of the product solution onto DNA tiling arrays and imaging the chip on a fluorescence scanner. (A) FAM-labeled IGFBP1 DNA treated for 0, 1, 2, 5 and 15 min with 0.2 units of exonuclease III at room temperature. (B) FAM-labeled IGFBP1 DNA treated for 0, 1, 2, 5 and 15 min with 2 units of exonuclease III at room temperature. (C) FAM-labeled IGFBP1 DNA treated for 0, 1, 2, 5 and 15 min with 20 units of exonuclease III at room temperature. The line profile directly below the tiling array images contains average intensities for the first 90 of 162 unique array features. Fluorescence signal from the remaining features was at background levels.
(TIF)

Figure S10 **Fragment length profile from digestion of FoxO1-IGFBP1 with exonuclease III as a function of time and enzyme dosage.** The digestion profile was visualized by application of the product solution onto DNA tiling arrays and imaging the chip on a fluorescence scanner. (A) FAM-labeled IGFBP1 DNA in complex with FoxO1 protein treated for 0, 1, 2, 5 and 15 min with 0.2 units of exonuclease III at room temperature. (B) Complex treated for 0, 1, 2, 5 and 15 min with 2 units of exonuclease III at room temperature. (C) Complex treated for 0, 1, 2, 5 and 15 min with 20 units of exonuclease III at room temperature. The line profile directly below the tiling array images contains average intensities for the first 90 of 162 unique array features. Fluorescence signal from the remaining features was at background levels.
(TIF)

Figure S11 **Fragment length profile from digestion of dsDNA with 0.2 units of exonuclease III as a function of time.** Exonuclease III was used to digest 100 ng FAM-labeled IGFBP1 DNA for 0, 1, 2, 5 and 15 min at room temperature. Exonuclease III digestions were stopped by addition of EDTA to a final concentration of 25 mM. The samples were subjected to fragment analysis using an ABI 3130xl Genetic Analyzer (Applied Biosystems, CA, USA).
(TIF)

Figure S12 **Fragment length profile from digestion of dsDNA with 2 units of exonuclease III as a function of time.** Exonuclease III was used to digest 100 ng FAM-labeled IGFBP1 DNA for 0, 1, 2, 5, and 15 min at room temperature. Exonuclease III digestions were stopped by addition of EDTA to a final concentration of 25 mM. The samples were then subjected to fragment analysis using an ABI 3130xl Genetic Analyzer (Applied Biosystems, CA, USA).
(TIF)

Figure S13 **Fragment length profile from digestion of dsDNA with 20 units of exonuclease III as a function of time.** Exonuclease III was used to digest 100 ng FAM-labeled IGFBP1 DNA for 0, 1, 2, 5, and 15 min at room temperature. Exonuclease III digestions were stopped by addition of EDTA to a final concentration of 25 mM. The samples were then subjected to fragment analysis using an ABI 3130xl Genetic Analyzer (Applied Biosystems, CA, USA).
(TIF)

Figure S14 **Fragment length profile from digestion of formaldehyde-treated FoxO1-IGFBP1 with 2 units of exonuclease III as a function of time and temperature.** Exonuclease III was used to digest 100 ng (DNA weight) FAM-labeled FoxO1-IGFBP1 complex pre-treated with 0.75% (v/v) formaldehyde for 0, 15, 45, and 120 min at 37°C as well as 45 min at room temperature. The digested complex was treated by proteinase K for 2 h at 65°C followed by cross-linking reversal in 250 mM Tris, pH 8.8, 0.5 M β-mercaptoethanol, and 2% SDS at 99°C for 25 min. The samples were then subjected to fragment analysis using an ABI 3130xl Genetic Analyzer (Applied Biosystems, CA, USA).
(TIF)

Figure S15 **Fragment length profile from digestion of formaldehyde-treated FoxO1-IGFBP1 with 20 units of exonuclease III as a function of time and temperature.** Exonuclease III was used to digest 100 ng (DNA weight) FAM-labeled FoxO1-IGFBP1 complex pre-treated with 0.75% (v/v) formaldehyde for 0, 15, 45, and 120 min at 37°C. The digested complex was treated by proteinase K for 2 h at 65°C followed by cross-linking reversal in 250 mM Tris, pH 8.8, 0.5 M β-mercaptoethanol, and 2% SDS at 99°C for 25 min. The samples were then subjected to fragment analysis using an ABI 3130xl Genetic Analyzer (Applied Biosystems, CA, USA).
(TIF)

Text S1 **Sequence of IGFBP1 promoter region (−25 to −204) PCR amplicon (5′→3′).** Character bordered sequences show the primers used for PCR amplification. Underlined sequences indicate FoxO1 binding sites including the FNBS (FoxO1 new binding site, 5′-ACAAACA-3′, described previously in Hatta et al. 2007) and two sites located in the IRE (insulin response element).
(PDF)

Table S1 **Target peptides for SRM analysis.**
(DOC)

Table S2 **The effects of cross-linking and cross-linking reversal on mass spectrometric analysis of the FoxO1-DNA complex using the SRM assay.**
(DOC)

Table S3 **Data Table for Discovery Mode Analysis of FoxO1 Protein Captured on Solid Supports Modified with Complementary and Non-Complementary (control) Capture Oligonucleotides.**
(DOC)

Table S4 **Oligonucleotide sequences on the DNA arrays.**
(DOC)
