# Supplementary figures and images for: Sequence-Specific Capture of Protein-DNA Complexes for Mass Spectrometric Protein Identification
Source: PLoS One. 2011 Oct 20;6(10):e26217. doi: 10.1371/journal.pone.0026217 (PMC3197616; doi:10.1371/journal.pone.0026217)

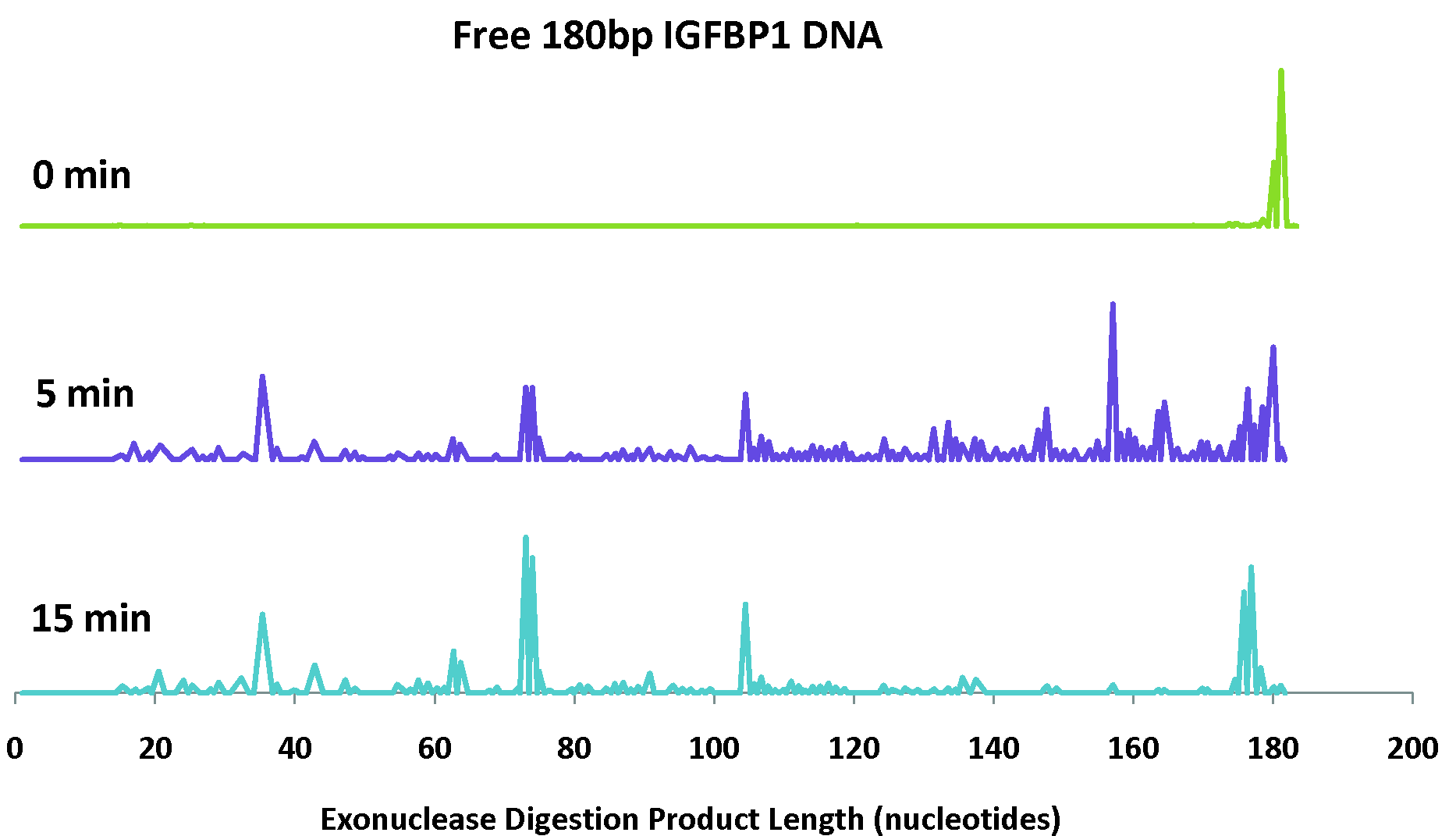

Supplement: Figure S1 — Fragment length profile from digestion of dsDNA with exonuclease III as a function of time. Two units of exonuclease III were used to digest 100 ng of FAM-labeled IGFBP1 DNA for 0, 5 and 15 min at room temperature. Exonuclease III digestions were stopped by addition of EDTA to a final concentration of 25 mM. The samples were subjected to fragment analysis using an ABI 3130xl Genetic Analyzer (Applied Biosystems, CA, USA). (TIF) [file pone.0026217.s001.tif]

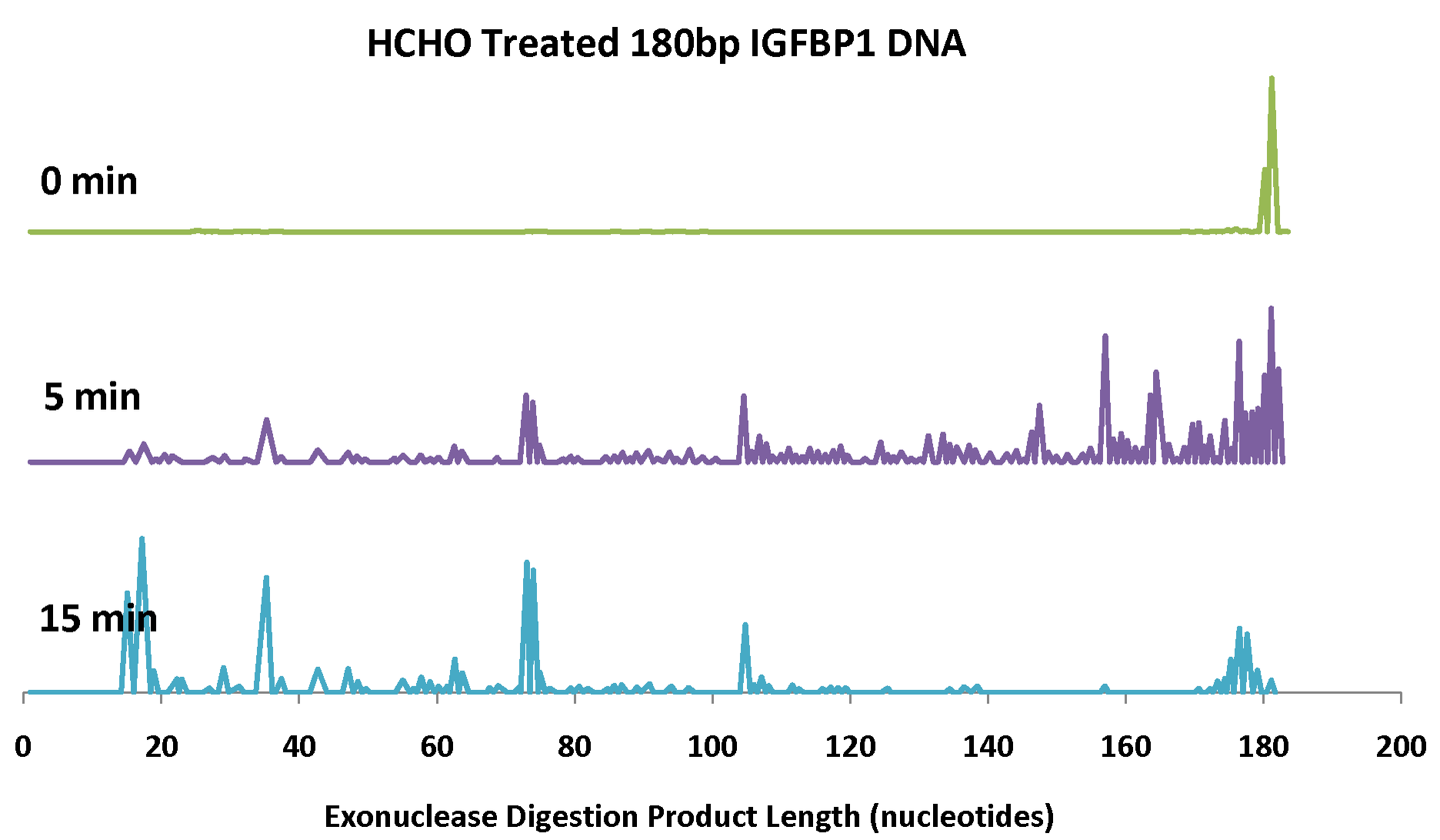

Supplement: Figure S2 — Fragment length profile from digestion of formaldehyde-treated dsDNA with exonuclease III as a function of time. FAM-labeled 180 bp IGFBP1 DNA was pretreated with 0.75% (v/v) formaldehyde for 10 min. The excess formaldehyde was diluted and buffer exchanged before exonuclease III digestion. Two units of exonuclease III were used to digest the DNA for 0, 5 and 15 min at room temperature. After digestion, the samples were subjected to fragment analysis using an ABI 3130xl Genetic Analyzer (Applied Biosystems, CA, USA). (TIF) [file pone.0026217.s002.tif]

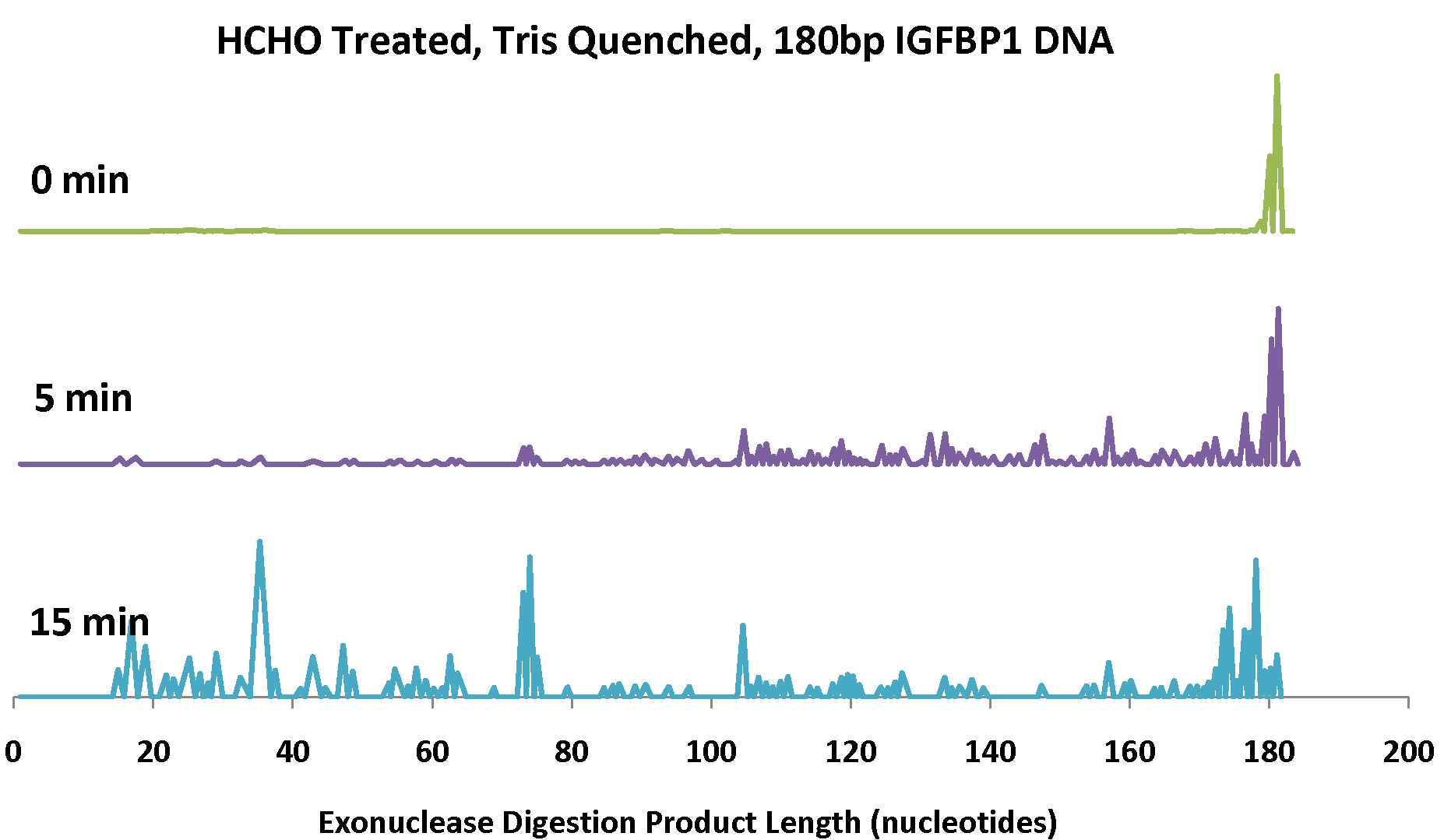

Supplement: Figure S3 — Fragment length profile from digestion of formaldehyde-treated and Tris-quenched dsDNA with exonuclease III as a function of time. The effect of cross-linking reagent (formaldehyde) and Tris quencher was evaluated by profiling the exonuclease digestion products. FAM-labeled 180 bp IGFBP1 DNA was pretreated with 0.75% (v/v) formaldehyde for 10 min, followed by quenching with 250 mM Tris. The formaldehyde and Tris were diluted and buffer exchanged. Two units of exonuclease III were used to digest the DNA for 0, 5 and 15 min at room temperature. After digestion, the samples were subjected to fragment analysis using an ABI 3130xl Genetic Analyzer (Applied Biosystems, CA, USA). (TIF) [file pone.0026217.s003.tif]

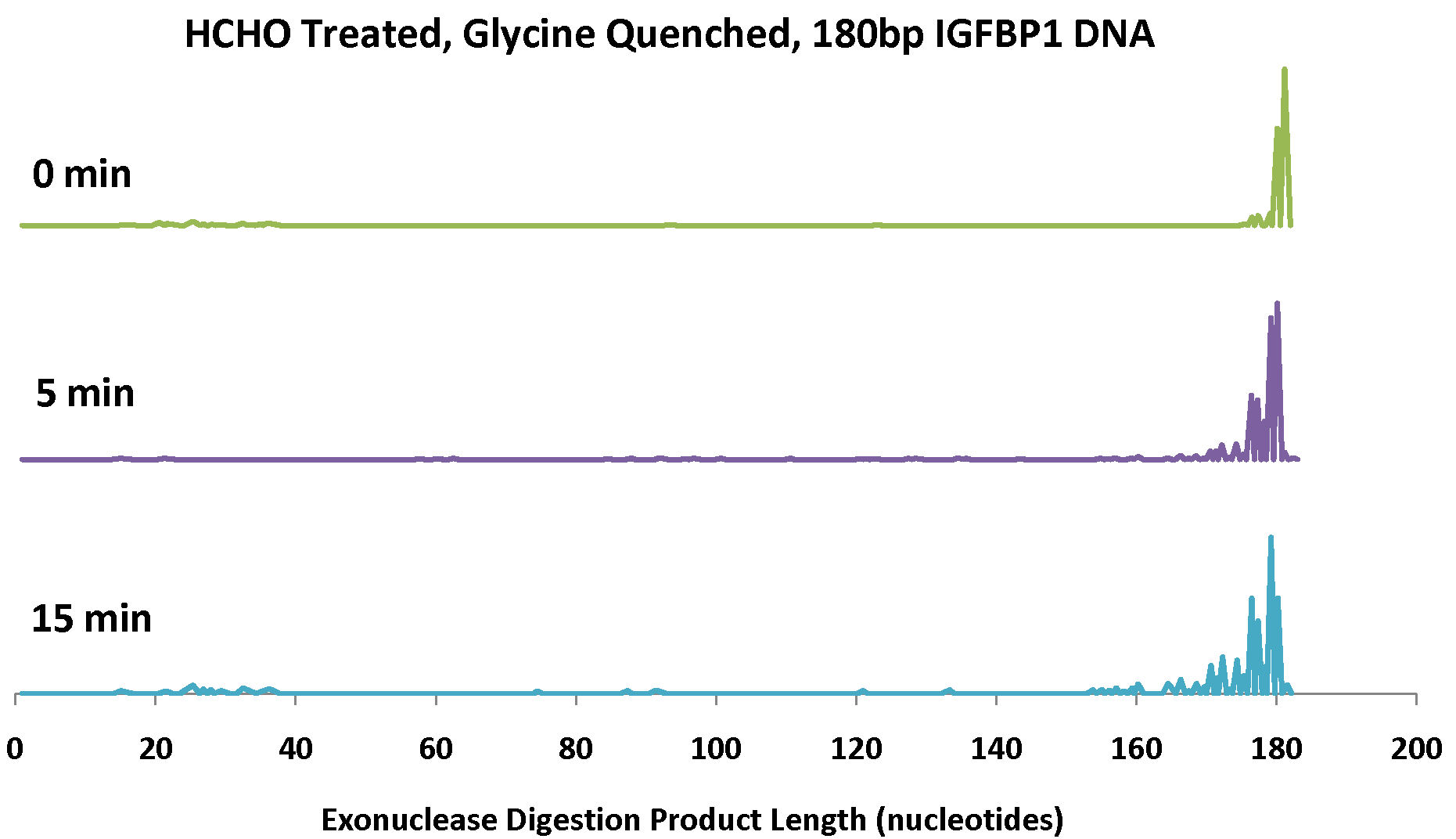

Supplement: Figure S4 — Fragment length profile from digestion of formaldehyde-treated and glycine-quenched dsDNA with exonuclease III as a function of time. The effect of cross-linking reagent (formaldehyde) and glycine quencher was evaluated by profiling the exonuclease digestion products. FAM-labeled 180 bp IGFBP1 DNA was pretreated with 0.75% (v/v) formaldehyde for 10 min, followed by quenching with 250 mM glycine. The formaldehyde and glycine were diluted and buffer exchanged. 2 units of exonuclease III were used to digest the DNA for 0, 5, and 15 min at room temperature. After digestion, the samples were subjected to fragment analysis using an ABI 3130xl Genetic Analyzer (Applied Biosystems, CA, USA). (TIF) [file pone.0026217.s004.tif]

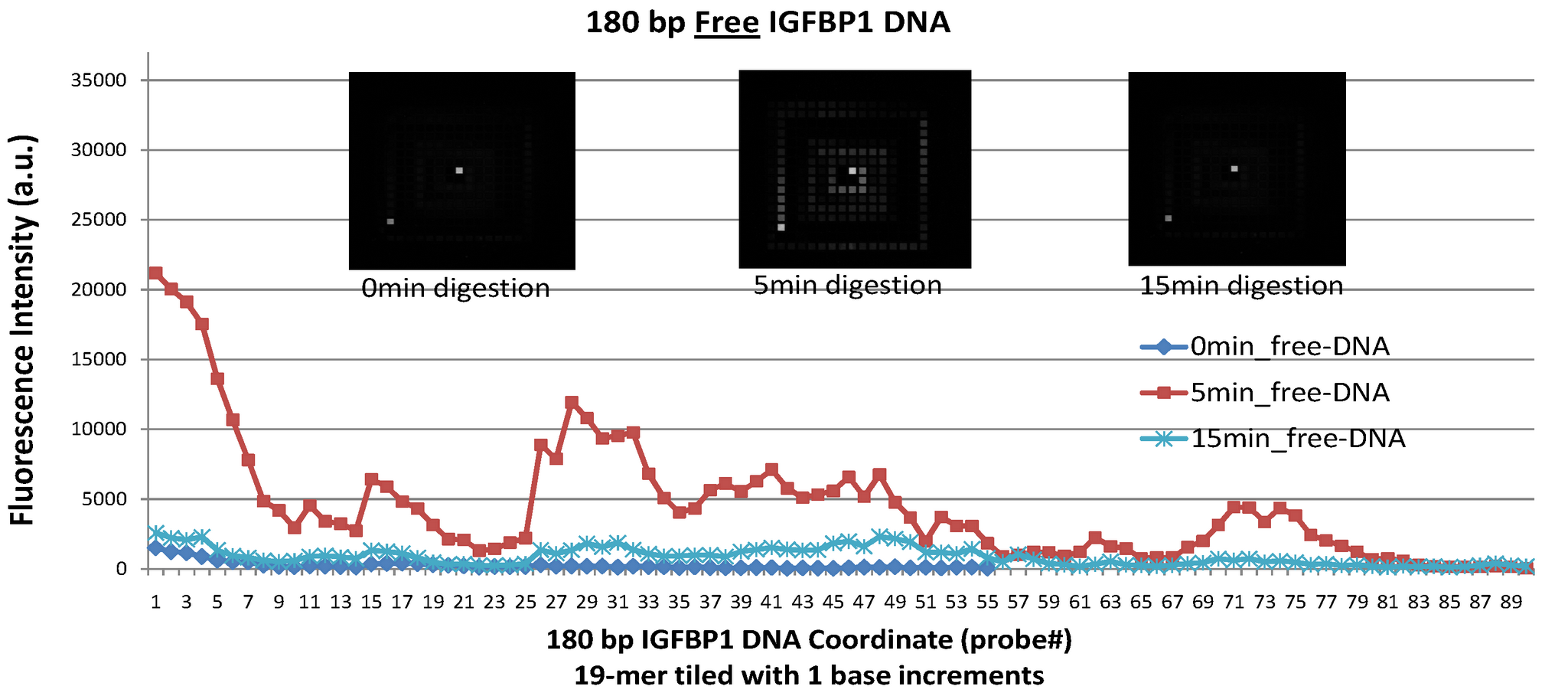

Supplement: Figure S5 — Fragment length profile from digestion of dsDNA with exonuclease III as a function of time. Two units of exonuclease III were used to digest FAM-labeled IGFBP1 DNA for 0, 5 and 15 min at room temperature. The digestion profile was visualized by application of the product solution onto DNA tiling arrays and imaging the substrate on a fluorescence scanner. The line profile directly below the tiling array images contains average intensities for the first 90 of 162 unique array features. Fluorescence signal from the remaining features was at background levels. (TIF) [file pone.0026217.s005.tif]

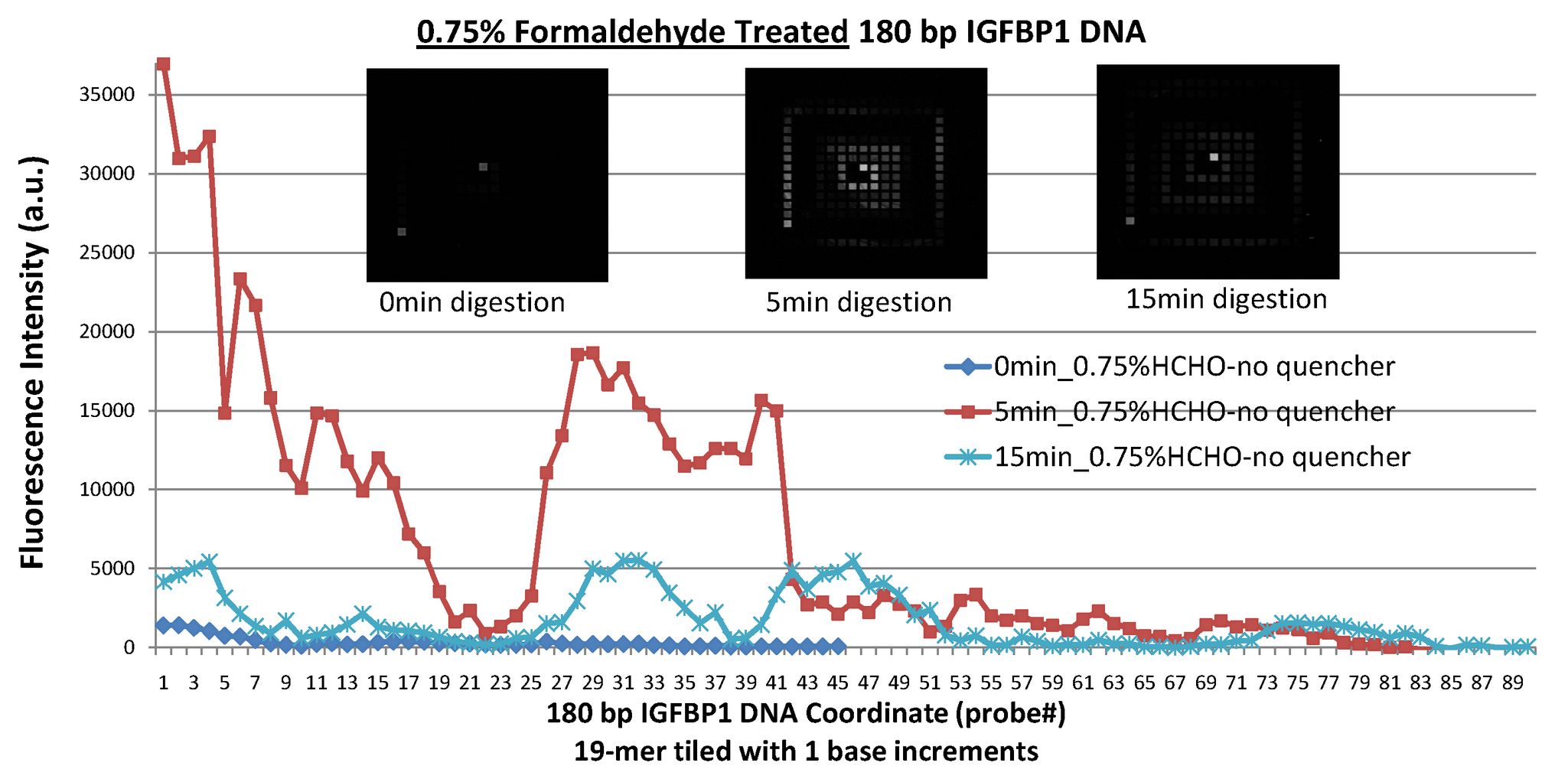

Supplement: Figure S6 — Fragment length profile from digestion of formaldehyde-treated dsDNA with exonuclease III as a function of time. FAM-labeled 180 bp IGFBP1 DNA was pretreated with 0.75% (v/v) formaldehyde for 10 min. The excess formaldehyde was diluted and buffer exchanged before exonuclease III digestion. Two units of exonuclease III were used to digest the DNA for 0, 5 and 15 min at room temperature. The digestion profile was visualized by application of the product solution onto DNA tiling arrays and imaging the substrate on a fluorescence scanner. The line profile directly below the tiling array images contains average intensities for the first 90 of 162 unique array features. Fluorescence signal from the remaining features was at background levels. (TIF) [file pone.0026217.s006.tif]

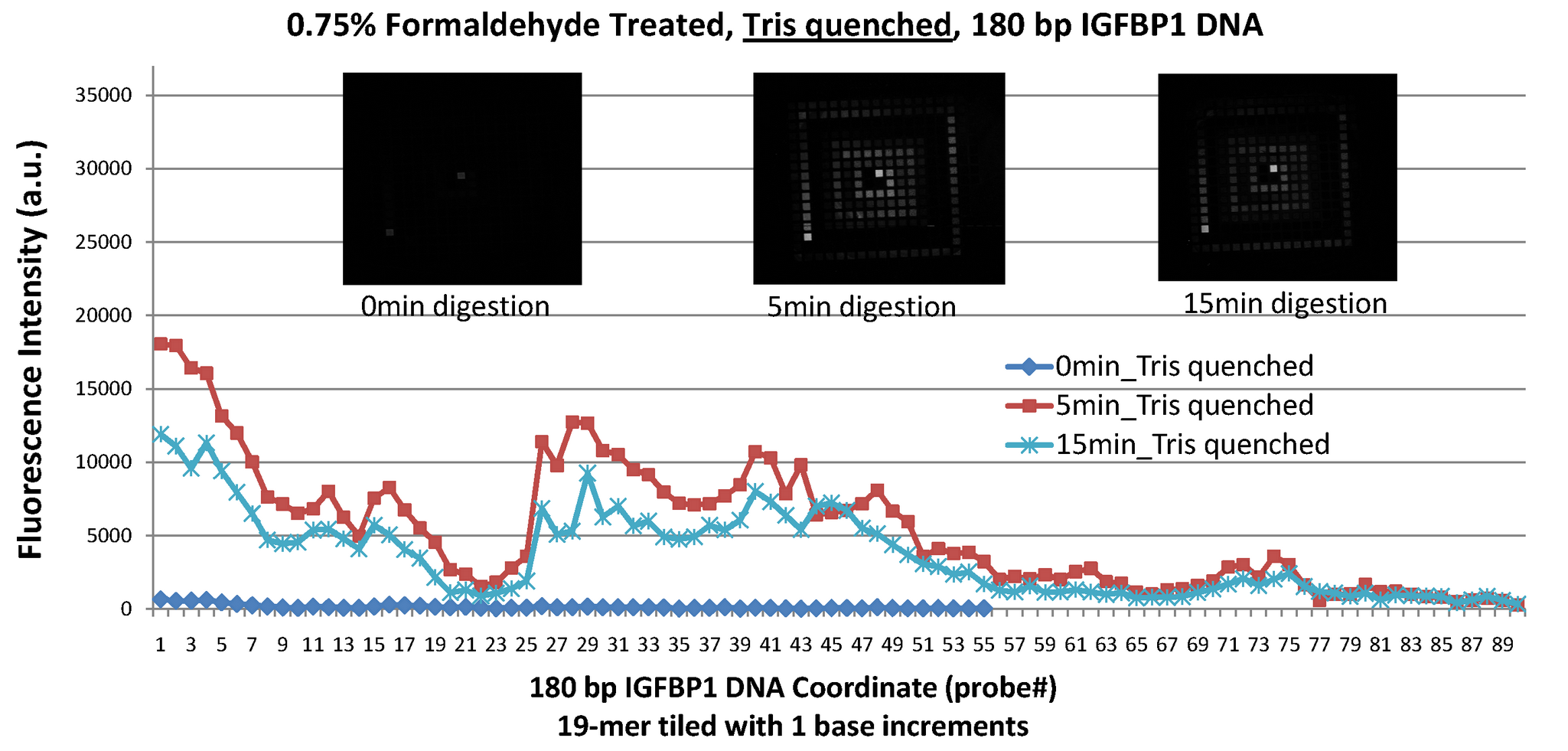

Supplement: Figure S7 — Fragment length profile from digestion of formaldehyde-treated and Tris-quenched dsDNA with exonuclease III as a function of time. FAM-labeled 180 bp IGFBP1 DNA was pretreated with 0.75% (v/v) formaldehyde for 10 min and quenched with 250 mM Tris. The formaldehyde and Tris were diluted and buffer exchanged before exonuclease III digestion. Two units of exonuclease III were used to digest the DNA for 0, 5 and 15 min at room temperature. The digestion profile was visualized by application of the product solution onto DNA tiling arrays and imaging the substrate on a fluorescence scanner. The line profile directly below the tiling array images contains average intensities for the first 90 of 162 unique array features. Fluorescence signal from the remaining features was at background levels. (TIF) [file pone.0026217.s007.tif]

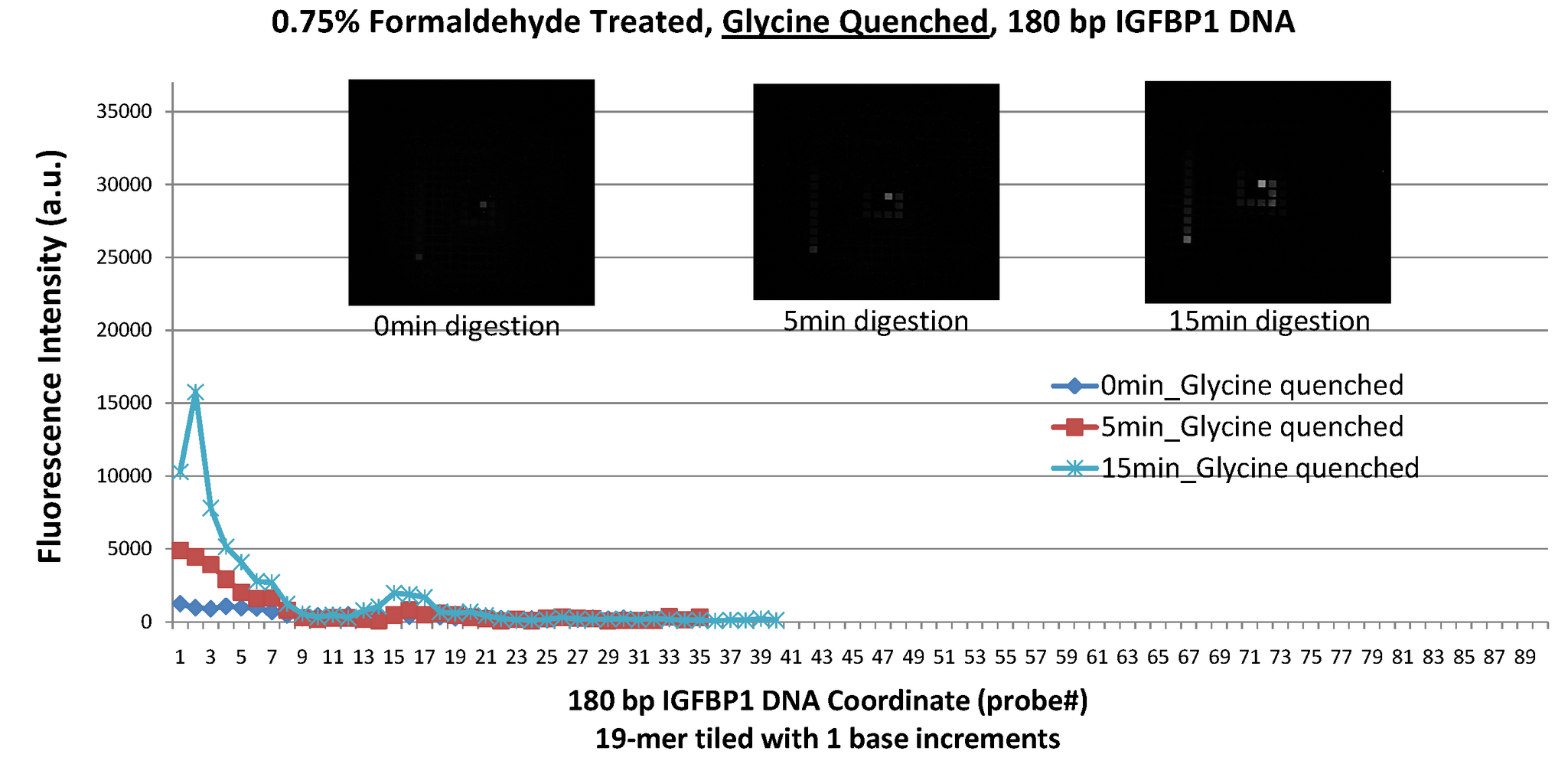

Supplement: Figure S8 — Fragment length profile from digestion of formaldehyde-treated and glycine-quenched dsDNA with exonuclease III as a function of time. FAM-labeled 180 bp IGFBP1 DNA was pretreated with 0.75% (v/v) formaldehyde for 10 min and quenched with 250 mM glycine. The formaldehyde and glycine were diluted and buffer exchanged before exonuclease III digestion. Two units of exonuclease III were used to digest the DNA for 0, 5 and 15 min at room temperature. The digestion profile was visualized by application of the product solution onto DNA tiling arrays and imaging the substrate on a fluorescence scanner. The line profile directly below the tiling array images contains average intensities for the first 90 of 162 unique array features. Fluorescence signal from the remaining features was at background levels. (TIF) [file pone.0026217.s008.tif]

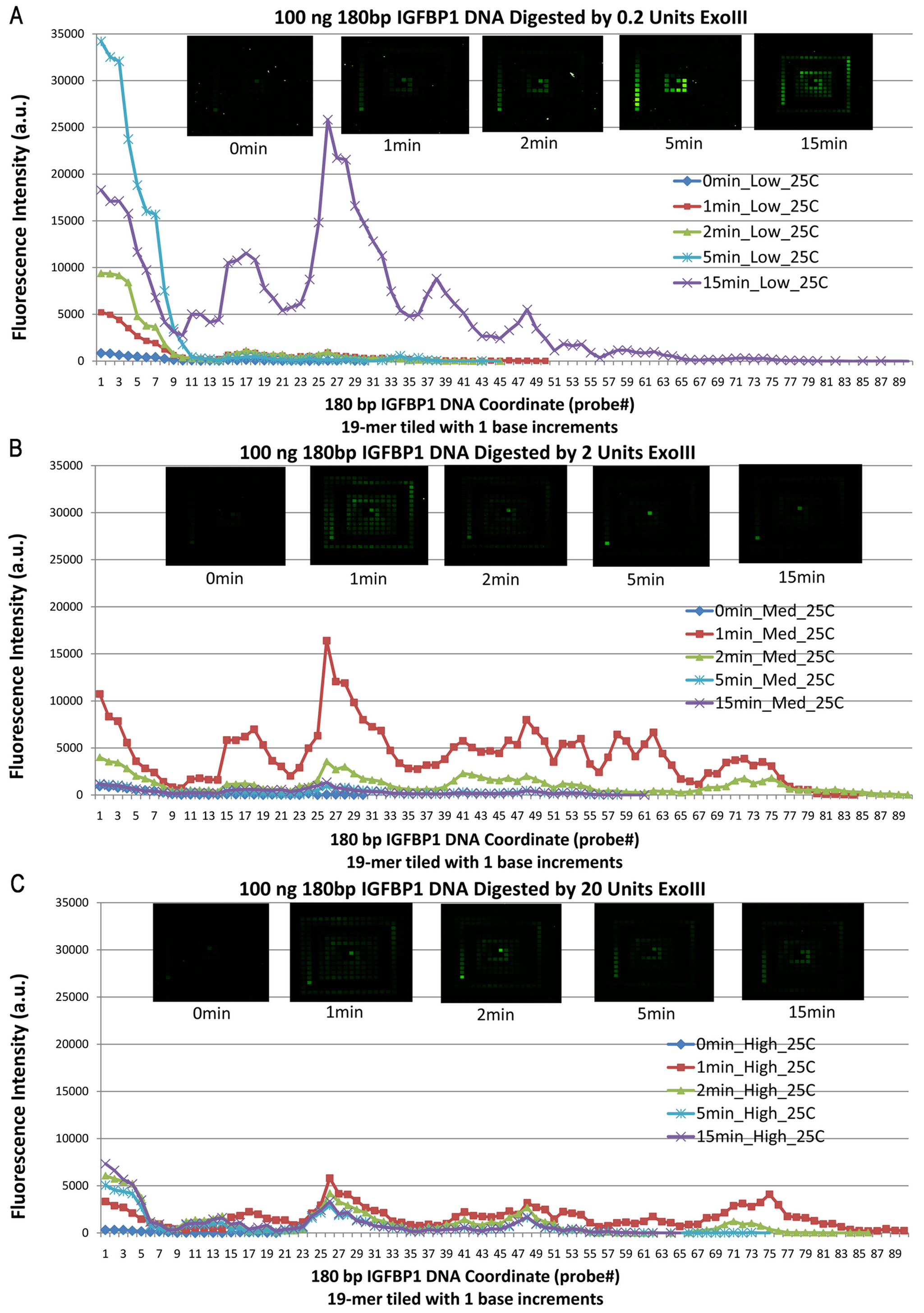

Supplement: Figure S9 — Fragment length profile from digestion of dsDNA with exonuclease III as a function of time and enzyme dosage. The digestion profile was visualized by application of the product solution onto DNA tiling arrays and imaging the chip on a fluorescence scanner. (A) FAM-labeled IGFBP1 DNA treated for 0, 1, 2, 5 and 15 min with 0.2 units of exonuclease III at room temperature. (B) FAM-labeled IGFBP1 DNA treated for 0, 1, 2, 5 and 15 min with 2 units of exonuclease III at room temperature. (C) FAM-labeled IGFBP1 DNA treated for 0, 1, 2, 5 and 15 min with 20 units of exonuclease III at room temperature. The line profile directly below the tiling array images contains average intensities for the first 90 of 162 unique array features. Fluorescence signal from the remaining features was at background levels. (TIF) [file pone.0026217.s009.tif]

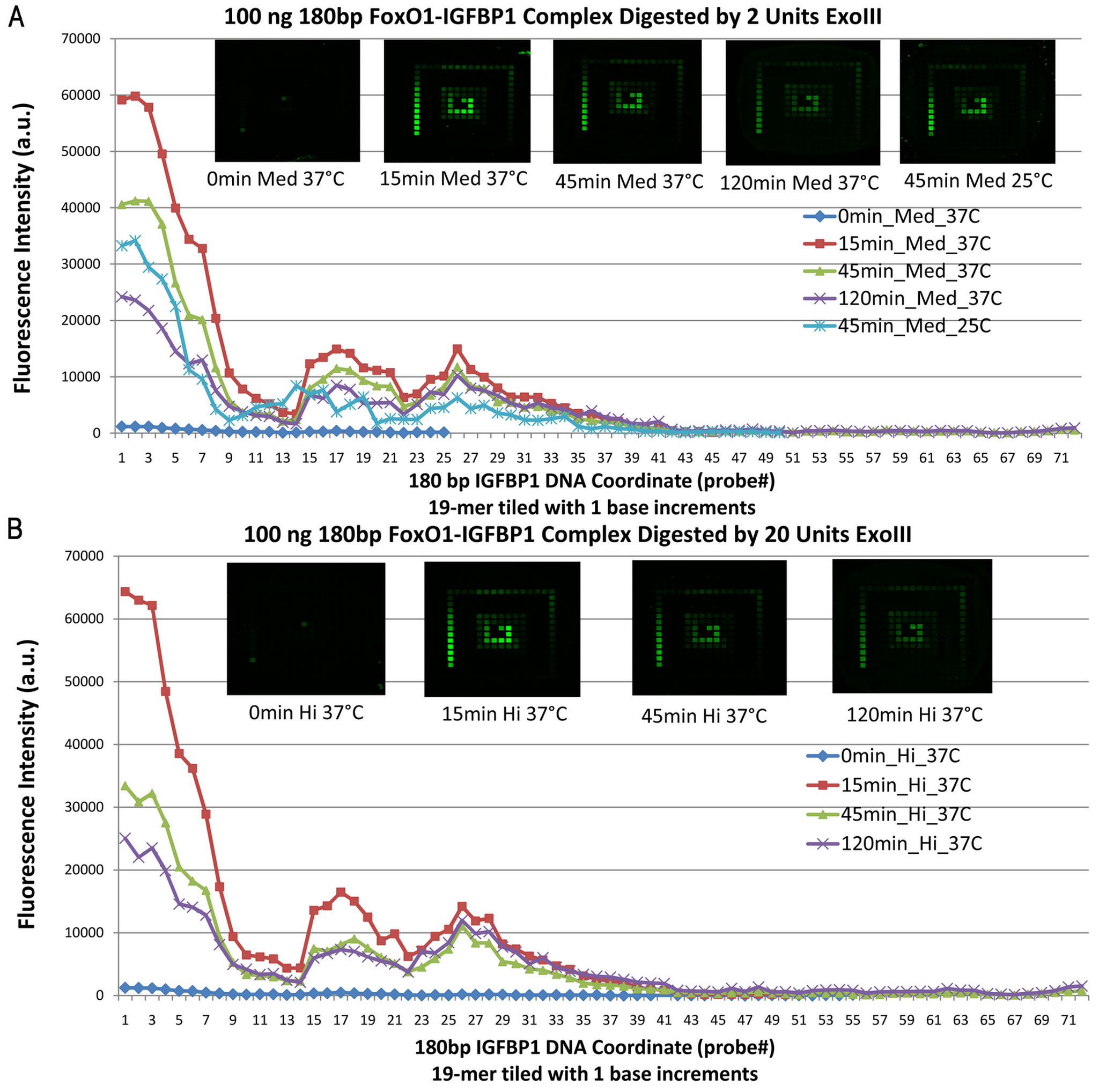

Supplement: Figure S10 — Fragment length profile from digestion of FoxO1-IGFBP1 with exonuclease III as a function of time and enzyme dosage. The digestion profile was visualized by application of the product solution onto DNA tiling arrays and imaging the chip on a fluorescence scanner. (A) FAM-labeled IGFBP1 DNA in complex with FoxO1 protein treated for 0, 1, 2, 5 and 15 min with 0.2 units of exonuclease III at room temperature. (B) Complex treated for 0, 1, 2, 5 and 15 min with 2 units of exonuclease III at room temperature. (C) Complex treated for 0, 1, 2, 5 and 15 min with 20 units of exonuclease III at room temperature. The line profile directly below the tiling array images contains average intensities for the first 90 of 162 unique array features. Fluorescence signal from the remaining features was at background levels. (TIF) [file pone.0026217.s010.tif]

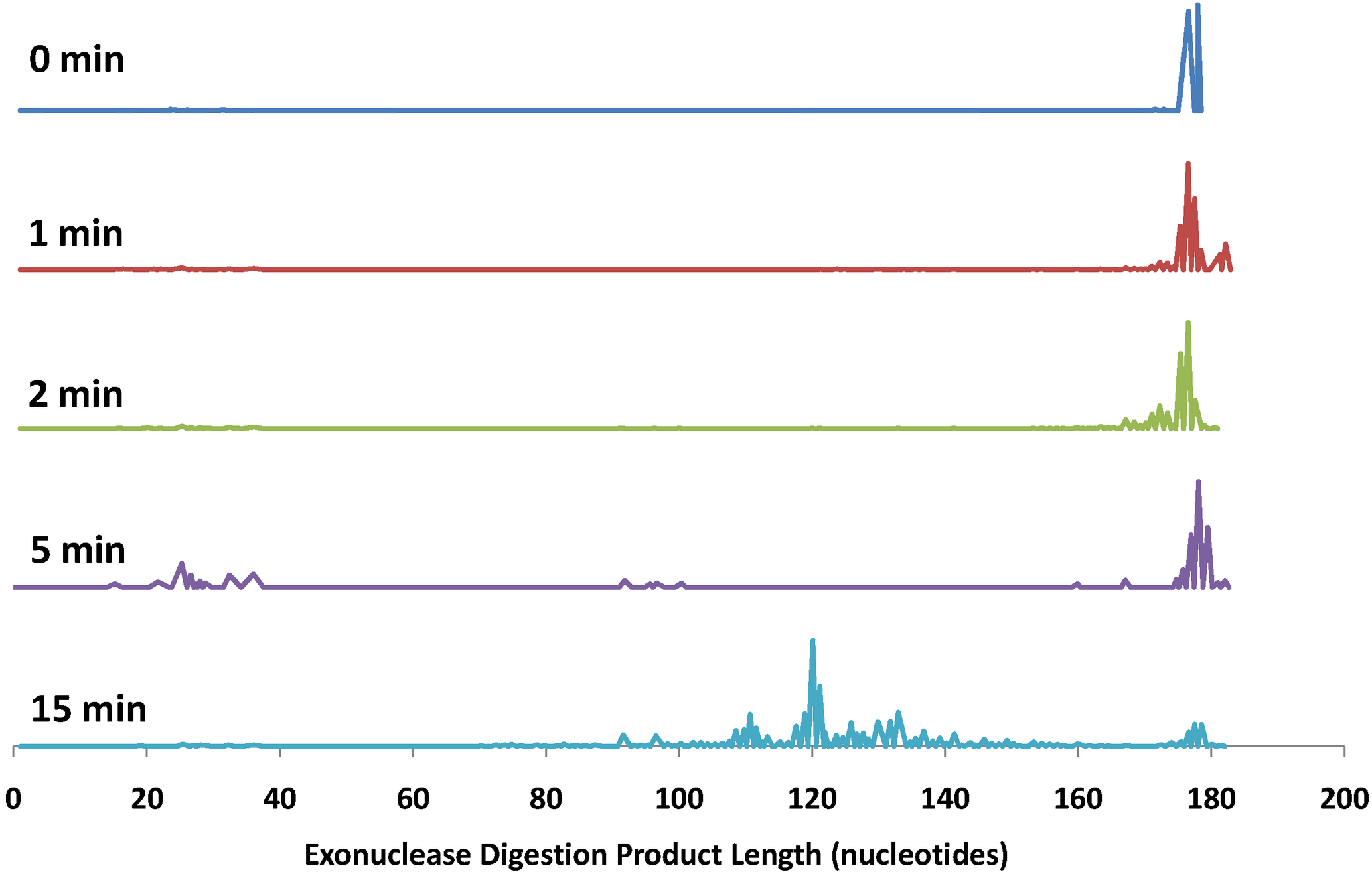

Supplement: Figure S11 — Fragment length profile from digestion of dsDNA with 0.2 units of exonuclease III as a function of time. Exonuclease III was used to digest 100 ng FAM-labeled IGFBP1 DNA for 0, 1, 2, 5 and 15 min at room temperature. Exonuclease III digestions were stopped by addition of EDTA to a final concentration of 25 mM. The samples were subjected to fragment analysis using an ABI 3130xl Genetic Analyzer (Applied Biosystems, CA, USA). (TIF) [file pone.0026217.s011.tif]

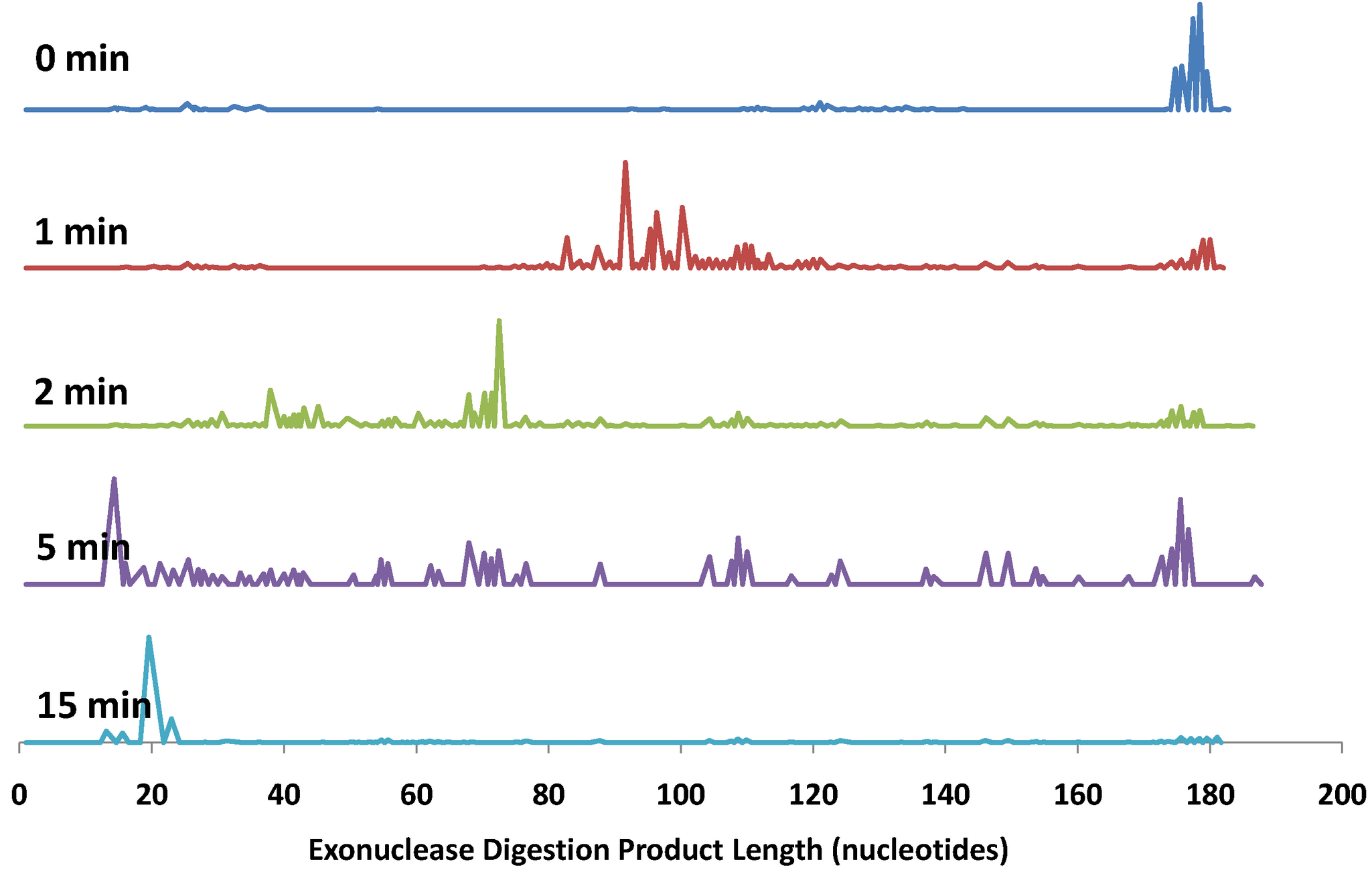

Supplement: Figure S12 — Fragment length profile from digestion of dsDNA with 2 units of exonuclease III as a function of time. Exonuclease III was used to digest 100 ng FAM-labeled IGFBP1 DNA for 0, 1, 2, 5, and 15 min at room temperature. Exonuclease III digestions were stopped by addition of EDTA to a final concentration of 25 mM. The samples were then subjected to fragment analysis using an ABI 3130xl Genetic Analyzer (Applied Biosystems, CA, USA). (TIF) [file pone.0026217.s012.tif]

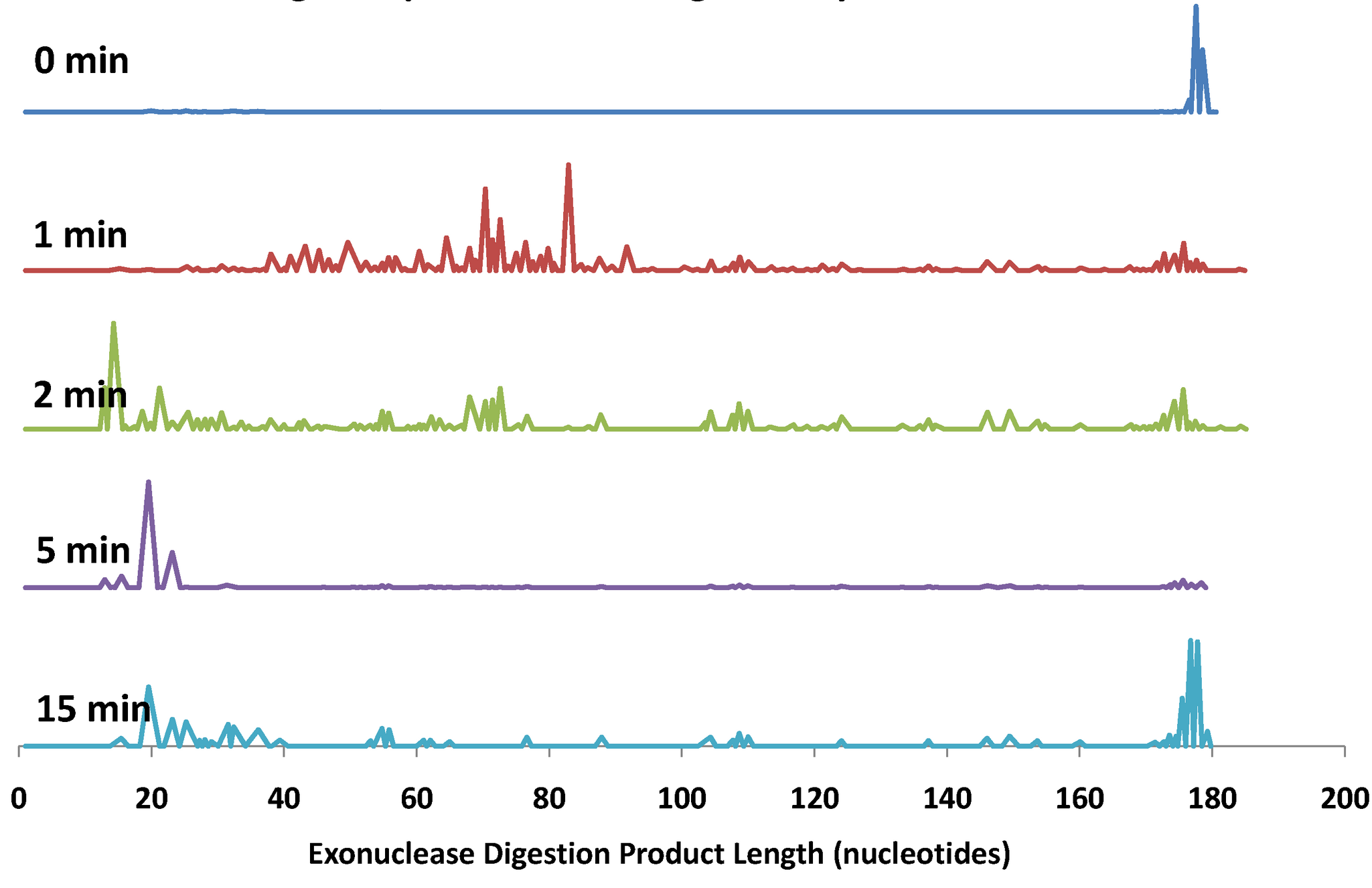

Supplement: Figure S13 — Fragment length profile from digestion of dsDNA with 20 units of exonuclease III as a function of time. Exonuclease III was used to digest 100 ng FAM-labeled IGFBP1 DNA for 0, 1, 2, 5, and 15 min at room temperature. Exonuclease III digestions were stopped by addition of EDTA to a final concentration of 25 mM. The samples were then subjected to fragment analysis using an ABI 3130xl Genetic Analyzer (Applied Biosystems, CA, USA). (TIF) [file pone.0026217.s013.tif]

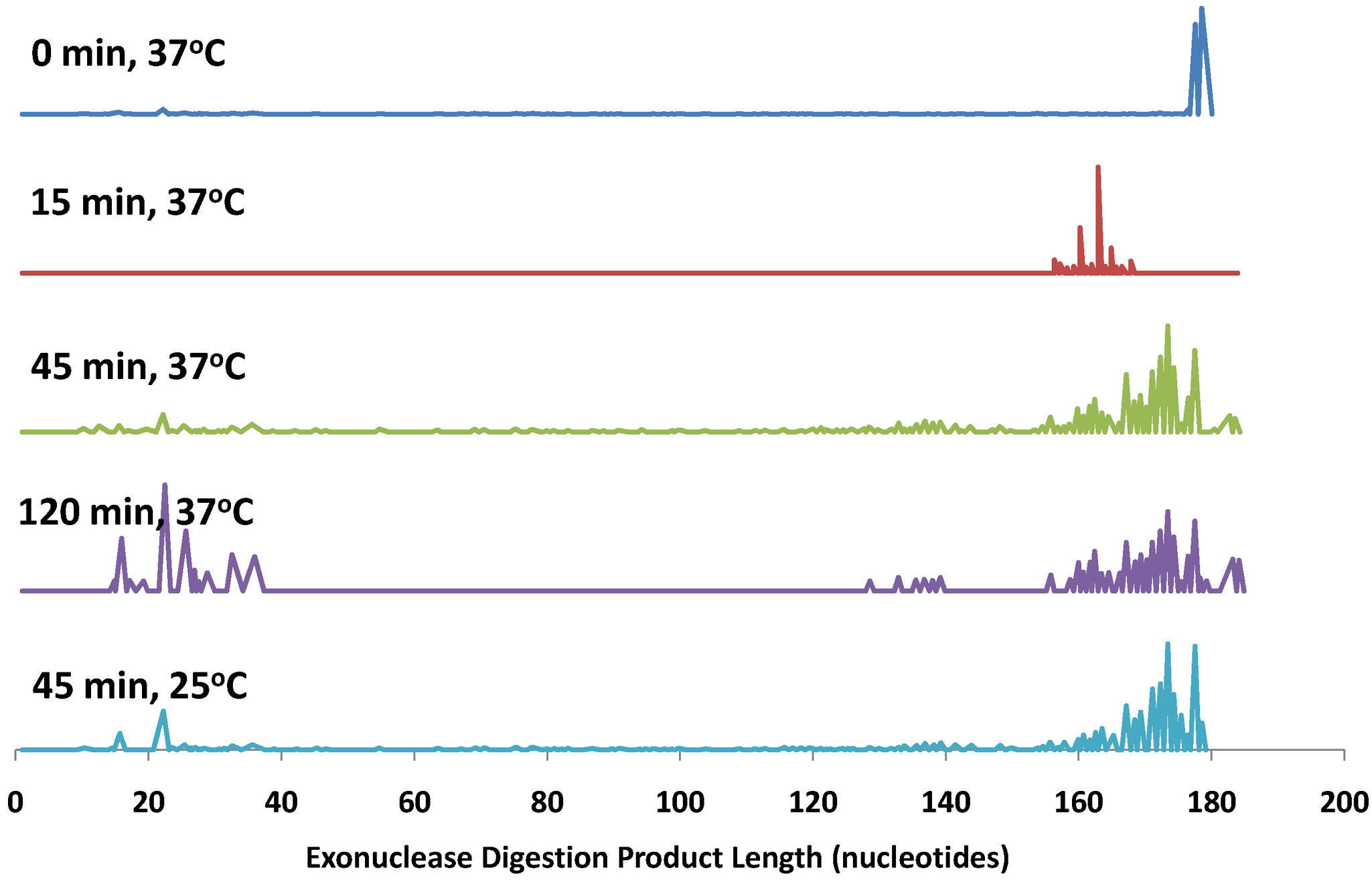

Supplement: Figure S14 — Fragment length profile from digestion of formaldehyde-treated FoxO1-IGFBP1 with 2 units of exonuclease III as a function of time and temperature. Exonuclease III was used to digest 100 ng (DNA weight) FAM-labeled FoxO1-IGFBP1 complex pre-treated with 0.75% (v/v) formaldehyde for 0, 15, 45, and 120 min at 37°C as well as 45 min at room temperature. The digested complex was treated by proteinase K for 2 h at 65°C followed by cross-linking reversal in 250 mM Tris, pH 8.8, 0.5 M β-mercaptoethanol, and 2% SDS at 99°C for 25 min. The samples were then subjected to fragment analysis using an ABI 3130xl Genetic Analyzer (Applied Biosystems, CA, USA). (TIF) [file pone.0026217.s014.tif]

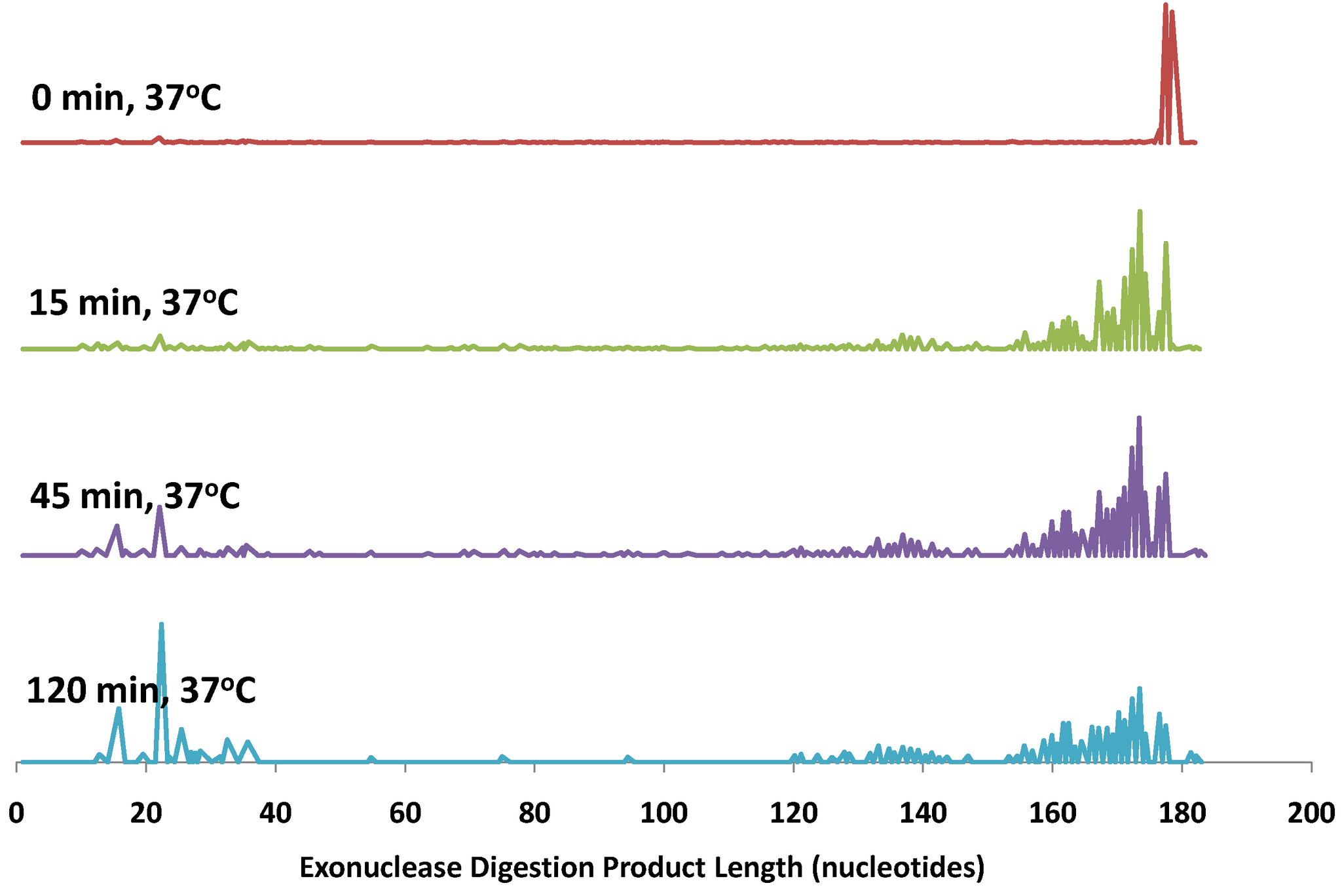

Supplement: Figure S15 — Fragment length profile from digestion of formaldehyde-treated FoxO1-IGFBP1 with 20 units of exonuclease III as a function of time and temperature. Exonuclease III was used to digest 100 ng (DNA weight) FAM-labeled FoxO1-IGFBP1 complex pre-treated with 0.75% (v/v) formaldehyde for 0, 15, 45, and 120 min at 37°C. The digested complex was treated by proteinase K for 2 h at 65°C followed by cross-linking reversal in 250 mM Tris, pH 8.8, 0.5 M β-mercaptoethanol, and 2% SDS at 99°C for 25 min. The samples were then subjected to fragment analysis using an ABI 3130xl Genetic Analyzer (Applied Biosystems, CA, USA). (TIF) [file pone.0026217.s015.tif]
