# Supplementary material for: Sequence-Specific Capture of Protein-DNA Complexes for Mass Spectrometric Protein Identification
Source: PLoS One. 2011 Oct 20;6(10):e26217. doi: 10.1371/journal.pone.0026217 (PMC3197616; doi:10.1371/journal.pone.0026217)
Supplement: Text S1 — Sequence of IGFBP1 promoter region (−25 to −204) PCR amplicon (5′→3′). Character bordered sequences show the primers used for PCR amplification. Underlined sequences indicate FoxO1 binding sites including the FNBS (FoxO1 new binding site, 5′-ACAAACA-3′, described previously in Hatta et al. 2007) and two sites located in the IRE (insulin response element). (PDF) [file pone.0026217.s016.pdf]

Text 1: Sequence of IGFBP1 promoter region (-25 to -204) PCR amplicon (5'→3'):

1 TTAGCTCCTG TCCCAGTCCA TCACCACGGG ACAAACATAG TAGAAAACCT

61 GATCCCTTTA CCCCCTTCCA CCCACGGTTT GTGTAGAGCT CACAAGCAAA

101 ACAAACTTAT TT*TGAACACT GGGGTCCT*AG CACGCTGCCC TGACAATCAT

161 TAACCTGTGC CGCACAGCCA GCCCTTCATA
