# Supplementary material for: Sequence-Specific Capture of Protein-DNA Complexes for Mass Spectrometric Protein Identification
Source: PLoS One. 2011 Oct 20;6(10):e26217. doi: 10.1371/journal.pone.0026217 (PMC3197616; doi:10.1371/journal.pone.0026217)
Supplement: Table S1 — Target peptides for SRM analysis. (DOC) [file pone.0026217.s017.doc]

**Table S**1. Target peptides for SRM analysis

| **Peptide Sequence** | **Isotopic Label** | **Precursor m/z** | **z** | **Product m/z (Transitions)** | | | **Collision Energy** | **Declustering Potential** |
| --- | --- | --- | --- | --- | --- | --- | --- | --- |
| NAWGNLSYADLITK | Light | 783.9 | 2.0 | 1010.0 | 1107.2 | 1164.2 | 40.4 | 88.3 |
|  | Heavy | 787.8 |  | 1018.0 | 1115.1 | 1172.2 |  |  |
| SVPYFK | Light | 370.9 | 2.0 | 457.5 | 554.7 | 653.8 | 16.9 | 58.2 |
|  | Heavy | 374.9 |  | 465.5 | 562.6 | 661.7 |  |  |
