# Supplementary material for: Sequence-Specific Capture of Protein-DNA Complexes for Mass Spectrometric Protein Identification
Source: PLoS One. 2011 Oct 20;6(10):e26217. doi: 10.1371/journal.pone.0026217 (PMC3197616; doi:10.1371/journal.pone.0026217)
Supplement: Table S2 — The effects of cross-linking and cross-linking reversal on mass spectrometric analysis of the FoxO1-DNA complex using the SRM assay. (DOC) [file pone.0026217.s018.doc]

**Table S2. The effects of cross-linking and cross-linking reversal on mass spectrometric analysis of the FoxO1-DNA complex using the SRM assay.**

| **Peptide** | | **NAWGNLSYADLITK** | | **SVPYFK** | |
| --- | --- | --- | --- | --- | --- |
|  |  | **(fmol)** | **Average** | **(fmol)** | **Average** |
| **Non-Cross-linked** | **Prep 1** | **32.40** | **32.74±0.76** | **61.04** | **61.60±0.69** |
|  |  | **33.02** |  | **61.85** |  |
|  |  | **33.30** |  | **61.25** |  |
|  | **Prep 2** | **31.33** |  | **61.02** |  |
|  |  | **33.23** |  | **61.56** |  |
|  |  | **33.14** |  | **62.85** |  |
| **Cross-linked** | **Prep 1** | **25.44** | **26.50±0.63** | **71.35** | **61.95±10.25** |
|  |  | **26.62** |  | **71.31** |  |
|  |  | **26.49** |  | **71.18** |  |
|  | **Prep 2** | **26.28** |  | **52.34** |  |
|  |  | **27.31** |  | **51.71** |  |
|  |  | **26.86** |  | **53.78** |  |
| **Cross-linking Reversed** | **Prep 1** | **0.63** | **0.58±0.19** | **4.15** | **5.22±1.10** |
|  |  | **0.84** |  | **4.22** |  |
|  |  | **0.38** |  | **4.34** |  |
|  | **Prep 2** | **0.76** |  | **5.98** |  |
|  |  | **0.42** |  | **6.05** |  |
|  |  | **0.44** |  | **6.58** |  |
