# Supplementary material for: Sequence-Specific Capture of Protein-DNA Complexes for Mass Spectrometric Protein Identification
Source: PLoS One. 2011 Oct 20;6(10):e26217. doi: 10.1371/journal.pone.0026217 (PMC3197616; doi:10.1371/journal.pone.0026217)
Supplement: Table S3 — Data Table for Discovery Mode Analysis of FoxO1 Protein Captured on Solid Supports Modified with Complementary and Non-Complementary (control) Capture Oligonucleotides. (DOC) [file pone.0026217.s019.doc]

**Table S3.** Data Table for Discovery Mode Analysis of FoxO1 Protein Captured on Solid Supports Modified with Complementary and Non-Complementary (control) Capture Oligonucleotides

| Thermo Velos-Orbitrap, Top 10 HCD fragmentation data dependent acquisition, filtered to 1% FDR rate | | | | | | | | | |
| --- | --- | --- | --- | --- | --- | --- | --- | --- | --- |
| FoxO1 Protein | **Sequence** | **Modifications** | **Probability** | **XCorr** | **Δ Score** | **Charge** | **m/z [Da]** | **MH+ [Da]** | **ΔM [ppm]** |
| Complementary DNA Chip Capture | RNAWGNLSYADLITK |  | 88.46 | 4.74 | 0.64 | 3 | 574.6361 | 1721.8938 | -1.88 |
|  | SVPYFKDKGDSNSSAGWK |  | 86.25 | 4.45 | 1 | 4 | 493.9909 | 1972.9417 | 0.84 |
|  | RNAWGNLSYADLITK |  | 92.89 | 4.28 | 0.68 | 3 | 574.637 | 1721.8963 | -0.39 |
|  | SVPYFKDKGDSNSSAGWK |  | 93.58 | 4.14 | 1 | 4 | 493.9899 | 1972.9376 | -1.2 |
|  | RLTLSQIYEWmVK | M11(Oxidation) | 57.95 | 4.06 | 1 | 3 | 561.6373 | 1682.8973 | 2.23 |
|  | LSPImTEQDDLGDGDVHSLVYPPSAAK | M5(Oxidation) | 83.35 | 4.04 | 1 | 3 | 957.7961 | 2871.3739 | 0.72 |
|  | RLTLSQIYEWMVK |  | 61.34 | 4.03 | 0.73 | 3 | 556.3051 | 1666.9006 | 1.21 |
|  | RLTLSQIYEWMVK |  | 44.14 | 3.95 | 0.71 | 3 | 556.3057 | 1666.9024 | 2.31 |
|  | LSPIMTEQDDLGDGDVHSLVYPPSAAK |  | 84.4 | 3.93 | 1 | 3 | 952.465 | 2855.3805 | 1.27 |
|  | LSPIMTEQDDLGDGDVHSLVYPPSAAK |  | 54.09 | 3.57 | 1 | 3 | 952.4652 | 2855.3811 | 1.46 |
|  | SSWWMLNPEGGK |  | 31.16 | 2.37 | 0.66 | 2 | 696.3234 | 1391.6395 | -1.34 |
| Non-complementary DNA Chip Capture | SVPYFKDKGDSNSSAGWK |  | 111.07 | 4.26 | 1 | 4 | 493.9905 | 1972.9403 | 0.16 |
|  | RNAWGNLSYADLITK |  | 83.65 | 4.26 | 1 | 3 | 574.6373 | 1721.8973 | 0.14 |
|  | RNAWGNLSYADLITK |  | 80.41 | 4.25 | 0.78 | 3 | 574.638 | 1721.8995 | 1.42 |
|  | LSPIMTEQDDLGDGDVHSLVYPPSAAK |  | 53.67 | 3.27 | 1 | 3 | 952.4656 | 2855.3822 | 1.85 |
|  | SSWWMLNPEGGK |  | 26.32 | 2.35 | 0.69 | 2 | 696.3254 | 1391.6436 | 1.64 |
