# Supplementary material for: Sequence-Specific Capture of Protein-DNA Complexes for Mass Spectrometric Protein Identification
Source: PLoS One. 2011 Oct 20;6(10):e26217. doi: 10.1371/journal.pone.0026217 (PMC3197616; doi:10.1371/journal.pone.0026217)
Supplement: Table S4 — Oligonucleotide sequences on the DNA arrays. (DOC) [file pone.0026217.s020.doc]

**Table S4. Oligonucleotide sequences on the DNA arrays**

| IGFBP1 capturing (complementary) and negative control (non-complementary) arrays. | | | | | | | |
| --- | --- | --- | --- | --- | --- | --- | --- |
| Name / Position | | | Sequence (3'-->5') |  | | |  |
| Capturing | | | TTTTTTTTTTTTTTTATACTTCCCGACCGACACG |  |  |  |  |
| Neg-Control | | | TTTTTTTTTTTTTTTCGGCTACTGGACGTTCTCA |  |  |  |  |
| IGFBP1 DNA tiling array oligonucleotide sequences.  (Note: The oligonucleotides were sequentially arranged on the tiling array in a clockwise order beginning at the center. The duplicate was arranged into the tiling array afterward.) | | | | | | | |
| Name / Position | | | Sequence (3'-->5') | Name / Position | | | Sequence (3'-->5') |
| Blank | | | T | 101 | to | 83 | TTTTTTTTTTTTTTTAAAACGAACACTCGAGATG |
| Control1 | | | TTTTTTTTTTTTTTTCGGCTACTGGACGTTCTCA | 100 | to | 82 | TTTTTTTTTTTTTTTAAACGAACACTCGAGATGT |
| Control2 | | | TTTTTTTTTTTTTTTTATTGAAACGTTGTCACC | 99 | to | 81 | TTTTTTTTTTTTTTTAACGAACACTCGAGATGTG |
| F-ctrl | | | TTTTTTTTTTTTTTTAATCGAGGACAGGGTCAGGT | 98 | to | 80 | TTTTTTTTTTTTTTTACGAACACTCGAGATGTGT |
| 180 | to | 162 | TTTTTTTTTTTTTTTATACTTCCCGACCGACACG | 97 | to | 79 | TTTTTTTTTTTTTTTCGAACACTCGAGATGTGTT |
| 180 | to | 162 | TTTTTTTTTTTTTTTATACTTCCCGACCGACACG | 96 | to | 78 | TTTTTTTTTTTTTTTGAACACTCGAGATGTGTTT |
| 179 | to | 161 | TTTTTTTTTTTTTTTTACTTCCCGACCGACACGC | 95 | to | 77 | TTTTTTTTTTTTTTTAACACTCGAGATGTGTTTG |
| 178 | to | 160 | TTTTTTTTTTTTTTTACTTCCCGACCGACACGCC | 94 | to | 76 | TTTTTTTTTTTTTTTACACTCGAGATGTGTTTGG |
| 177 | to | 159 | TTTTTTTTTTTTTTTCTTCCCGACCGACACGCCG | 93 | to | 75 | TTTTTTTTTTTTTTTCACTCGAGATGTGTTTGGC |
| 176 | to | 158 | TTTTTTTTTTTTTTTTTCCCGACCGACACGCCGT | 92 | to | 74 | TTTTTTTTTTTTTTTACTCGAGATGTGTTTGGCA |
| 175 | to | 157 | TTTTTTTTTTTTTTTTCCCGACCGACACGCCGTG | 91 | to | 73 | TTTTTTTTTTTTTTTCTCGAGATGTGTTTGGCAC |
| 174 | to | 156 | TTTTTTTTTTTTTTTCCCGACCGACACGCCGTGT | 90 | to | 72 | TTTTTTTTTTTTTTTTCGAGATGTGTTTGGCACC |
| 173 | to | 155 | TTTTTTTTTTTTTTTCCGACCGACACGCCGTGTC | 89 | to | 71 | TTTTTTTTTTTTTTTCGAGATGTGTTTGGCACCC |
| 172 | to | 154 | TTTTTTTTTTTTTTTCGACCGACACGCCGTGTCC | 88 | to | 70 | TTTTTTTTTTTTTTTGAGATGTGTTTGGCACCCA |
| 171 | to | 153 | TTTTTTTTTTTTTTTGACCGACACGCCGTGTCCA | 87 | to | 69 | TTTTTTTTTTTTTTTAGATGTGTTTGGCACCCAC |
| 170 | to | 152 | TTTTTTTTTTTTTTTACCGACACGCCGTGTCCAA | 86 | to | 68 | TTTTTTTTTTTTTTTGATGTGTTTGGCACCCACC |
| 169 | to | 151 | TTTTTTTTTTTTTTTCCGACACGCCGTGTCCAAT | 85 | to | 67 | TTTTTTTTTTTTTTTATGTGTTTGGCACCCACCT |
| 168 | to | 150 | TTTTTTTTTTTTTTTCGACACGCCGTGTCCAATT | 84 | to | 66 | TTTTTTTTTTTTTTTTGTGTTTGGCACCCACCTT |
| 167 | to | 149 | TTTTTTTTTTTTTTTGACACGCCGTGTCCAATTA | 83 | to | 65 | TTTTTTTTTTTTTTTGTGTTTGGCACCCACCTTC |
| 166 | to | 148 | TTTTTTTTTTTTTTTACACGCCGTGTCCAATTAC | 82 | to | 64 | TTTTTTTTTTTTTTTTGTTTGGCACCCACCTTCC |
| 165 | to | 147 | TTTTTTTTTTTTTTTCACGCCGTGTCCAATTACT | 81 | to | 63 | TTTTTTTTTTTTTTTGTTTGGCACCCACCTTCCC |
| 164 | to | 146 | TTTTTTTTTTTTTTTACGCCGTGTCCAATTACTA | 80 | to | 62 | TTTTTTTTTTTTTTTTTTGGCACCCACCTTCCCC |
| 163 | to | 145 | TTTTTTTTTTTTTTTCGCCGTGTCCAATTACTAA | 79 | to | 61 | TTTTTTTTTTTTTTTTTGGCACCCACCTTCCCCC |
| 162 | to | 144 | TTTTTTTTTTTTTTTGCCGTGTCCAATTACTAAC | 78 | to | 60 | TTTTTTTTTTTTTTTTGGCACCCACCTTCCCCCA |
| 161 | to | 143 | TTTTTTTTTTTTTTTCCGTGTCCAATTACTAACA | 77 | to | 59 | TTTTTTTTTTTTTTTGGCACCCACCTTCCCCCAT |
| 160 | to | 142 | TTTTTTTTTTTTTTTCGTGTCCAATTACTAACAG | 76 | to | 58 | TTTTTTTTTTTTTTTGCACCCACCTTCCCCCATT |
| 159 | to | 141 | TTTTTTTTTTTTTTTGTGTCCAATTACTAACAGT | 75 | to | 57 | TTTTTTTTTTTTTTTCACCCACCTTCCCCCATTT |
| 158 | to | 140 | TTTTTTTTTTTTTTTTGTCCAATTACTAACAGTC | 74 | to | 56 | TTTTTTTTTTTTTTTACCCACCTTCCCCCATTTC |
| 157 | to | 139 | TTTTTTTTTTTTTTTGTCCAATTACTAACAGTCC | 73 | to | 55 | TTTTTTTTTTTTTTTCCCACCTTCCCCCATTTCC |
| 156 | to | 138 | TTTTTTTTTTTTTTTTCCAATTACTAACAGTCCC | 72 | to | 54 | TTTTTTTTTTTTTTTCCACCTTCCCCCATTTCCC |
| 155 | to | 137 | TTTTTTTTTTTTTTTCCAATTACTAACAGTCCCG | 71 | to | 53 | TTTTTTTTTTTTTTTCACCTTCCCCCATTTCCCT |
| 154 | to | 136 | TTTTTTTTTTTTTTTCAATTACTAACAGTCCCGT | 70 | to | 52 | TTTTTTTTTTTTTTTACCTTCCCCCATTTCCCTA |
| 153 | to | 135 | TTTTTTTTTTTTTTTAATTACTAACAGTCCCGTC | 69 | to | 51 | TTTTTTTTTTTTTTTCCTTCCCCCATTTCCCTAG |
| 152 | to | 134 | TTTTTTTTTTTTTTTATTACTAACAGTCCCGTCG | 68 | to | 50 | TTTTTTTTTTTTTTTCTTCCCCCATTTCCCTAGT |
| 151 | to | 133 | TTTTTTTTTTTTTTTTTACTAACAGTCCCGTCGC | 67 | to | 49 | TTTTTTTTTTTTTTTTTCCCCCATTTCCCTAGTC |
| 150 | to | 132 | TTTTTTTTTTTTTTTTACTAACAGTCCCGTCGCA | 66 | to | 48 | TTTTTTTTTTTTTTTTCCCCCATTTCCCTAGTCC |
| 149 | to | 131 | TTTTTTTTTTTTTTTACTAACAGTCCCGTCGCAC | 65 | to | 47 | TTTTTTTTTTTTTTTCCCCCATTTCCCTAGTCCA |
| 148 | to | 130 | TTTTTTTTTTTTTTTCTAACAGTCCCGTCGCACG | 64 | to | 46 | TTTTTTTTTTTTTTTCCCCATTTCCCTAGTCCAA |
| 147 | to | 129 | TTTTTTTTTTTTTTTTAACAGTCCCGTCGCACGA | 63 | to | 45 | TTTTTTTTTTTTTTTCCCATTTCCCTAGTCCAAA |
| 146 | to | 128 | TTTTTTTTTTTTTTTAACAGTCCCGTCGCACGAT | 62 | to | 44 | TTTTTTTTTTTTTTTCCATTTCCCTAGTCCAAAA |
| 145 | to | 127 | TTTTTTTTTTTTTTTACAGTCCCGTCGCACGATC | 61 | to | 43 | TTTTTTTTTTTTTTTCATTTCCCTAGTCCAAAAG |
| 144 | to | 126 | TTTTTTTTTTTTTTTCAGTCCCGTCGCACGATCC | 60 | to | 42 | TTTTTTTTTTTTTTTATTTCCCTAGTCCAAAAGA |
| 143 | to | 125 | TTTTTTTTTTTTTTTAGTCCCGTCGCACGATCCT | 59 | to | 41 | TTTTTTTTTTTTTTTTTTCCCTAGTCCAAAAGAT |
| 142 | to | 124 | TTTTTTTTTTTTTTTGTCCCGTCGCACGATCCTG | 58 | to | 40 | TTTTTTTTTTTTTTTTTCCCTAGTCCAAAAGATG |
| 141 | to | 123 | TTTTTTTTTTTTTTTTCCCGTCGCACGATCCTGG | 57 | to | 39 | TTTTTTTTTTTTTTTTCCCTAGTCCAAAAGATGA |
| 140 | to | 122 | TTTTTTTTTTTTTTTCCCGTCGCACGATCCTGGG | 56 | to | 38 | TTTTTTTTTTTTTTTCCCTAGTCCAAAAGATGAT |
| 139 | to | 121 | TTTTTTTTTTTTTTTCCGTCGCACGATCCTGGGG | 55 | to | 37 | TTTTTTTTTTTTTTTCCTAGTCCAAAAGATGATA |
| 138 | to | 120 | TTTTTTTTTTTTTTTCGTCGCACGATCCTGGGGT | 54 | to | 36 | TTTTTTTTTTTTTTTCTAGTCCAAAAGATGATAC |
| 137 | to | 119 | TTTTTTTTTTTTTTTGTCGCACGATCCTGGGGTC | 53 | to | 35 | TTTTTTTTTTTTTTTTAGTCCAAAAGATGATACA |
| 136 | to | 118 | TTTTTTTTTTTTTTTTCGCACGATCCTGGGGTCA | 52 | to | 34 | TTTTTTTTTTTTTTTAGTCCAAAAGATGATACAA |
| 135 | to | 117 | TTTTTTTTTTTTTTTCGCACGATCCTGGGGTCAC | 51 | to | 33 | TTTTTTTTTTTTTTTGTCCAAAAGATGATACAAA |
| 134 | to | 116 | TTTTTTTTTTTTTTTGCACGATCCTGGGGTCACA | 50 | to | 32 | TTTTTTTTTTTTTTTTCCAAAAGATGATACAAAC |
| 133 | to | 115 | TTTTTTTTTTTTTTTCACGATCCTGGGGTCACAA | 49 | to | 31 | TTTTTTTTTTTTTTTCCAAAAGATGATACAAACA |
| 132 | to | 114 | TTTTTTTTTTTTTTTACGATCCTGGGGTCACAAG | 48 | to | 30 | TTTTTTTTTTTTTTTCAAAAGATGATACAAACAG |
| 131 | to | 113 | TTTTTTTTTTTTTTTCGATCCTGGGGTCACAAGT | 47 | to | 29 | TTTTTTTTTTTTTTTAAAAGATGATACAAACAGG |
| 130 | to | 112 | TTTTTTTTTTTTTTTGATCCTGGGGTCACAAGTT | 46 | to | 28 | TTTTTTTTTTTTTTTAAAGATGATACAAACAGGG |
| 129 | to | 111 | TTTTTTTTTTTTTTTATCCTGGGGTCACAAGTTT | 45 | to | 27 | TTTTTTTTTTTTTTTAAGATGATACAAACAGGGC |
| 128 | to | 110 | TTTTTTTTTTTTTTTTCCTGGGGTCACAAGTTTT | 44 | to | 26 | TTTTTTTTTTTTTTTAGATGATACAAACAGGGCA |
| 127 | to | 109 | TTTTTTTTTTTTTTTCCTGGGGTCACAAGTTTTA | 43 | to | 25 | TTTTTTTTTTTTTTTGATGATACAAACAGGGCAC |
| 126 | to | 108 | TTTTTTTTTTTTTTTCTGGGGTCACAAGTTTTAT | 42 | to | 24 | TTTTTTTTTTTTTTTATGATACAAACAGGGCACC |
| 125 | to | 107 | TTTTTTTTTTTTTTTTGGGGTCACAAGTTTTATT | 41 | to | 23 | TTTTTTTTTTTTTTTTGATACAAACAGGGCACCA |
| 124 | to | 106 | TTTTTTTTTTTTTTTGGGGTCACAAGTTTTATTC | 40 | to | 22 | TTTTTTTTTTTTTTTGATACAAACAGGGCACCAC |
| 123 | to | 105 | TTTTTTTTTTTTTTTGGGTCACAAGTTTTATTCA | 39 | to | 21 | TTTTTTTTTTTTTTTATACAAACAGGGCACCACT |
| 122 | to | 104 | TTTTTTTTTTTTTTTGGTCACAAGTTTTATTCAA | 38 | to | 20 | TTTTTTTTTTTTTTTTACAAACAGGGCACCACTA |
| 121 | to | 103 | TTTTTTTTTTTTTTTGTCACAAGTTTTATTCAAA | 37 | to | 19 | TTTTTTTTTTTTTTTACAAACAGGGCACCACTAC |
| 120 | to | 102 | TTTTTTTTTTTTTTTTCACAAGTTTTATTCAAAC | 36 | to | 18 | TTTTTTTTTTTTTTTCAAACAGGGCACCACTACC |
| 119 | to | 101 | TTTTTTTTTTTTTTTCACAAGTTTTATTCAAACA | 35 | to | 17 | TTTTTTTTTTTTTTTAAACAGGGCACCACTACCT |
| 118 | to | 100 | TTTTTTTTTTTTTTTACAAGTTTTATTCAAACAA | 34 | to | 16 | TTTTTTTTTTTTTTTAACAGGGCACCACTACCTG |
| 117 | to | 99 | TTTTTTTTTTTTTTTCAAGTTTTATTCAAACAAA | 33 | to | 15 | TTTTTTTTTTTTTTTACAGGGCACCACTACCTGA |
| 116 | to | 98 | TTTTTTTTTTTTTTTAAGTTTTATTCAAACAAAA | 32 | to | 14 | TTTTTTTTTTTTTTTCAGGGCACCACTACCTGAC |
| 115 | to | 97 | TTTTTTTTTTTTTTTAGTTTTATTCAAACAAAAC | 31 | to | 13 | TTTTTTTTTTTTTTTAGGGCACCACTACCTGACC |
| 114 | to | 96 | TTTTTTTTTTTTTTTGTTTTATTCAAACAAAACG | 30 | to | 12 | TTTTTTTTTTTTTTTGGGCACCACTACCTGACCC |
| 113 | to | 95 | TTTTTTTTTTTTTTTTTTTATTCAAACAAAACGA | 29 | to | 11 | TTTTTTTTTTTTTTTGGCACCACTACCTGACCCT |
| 112 | to | 94 | TTTTTTTTTTTTTTTTTTATTCAAACAAAACGAA | 28 | to | 10 | TTTTTTTTTTTTTTTGCACCACTACCTGACCCTG |
| 111 | to | 93 | TTTTTTTTTTTTTTTTTATTCAAACAAAACGAAC | 27 | to | 9 | TTTTTTTTTTTTTTTCACCACTACCTGACCCTGT |
| 110 | to | 92 | TTTTTTTTTTTTTTTTATTCAAACAAAACGAACA | 26 | to | 8 | TTTTTTTTTTTTTTTACCACTACCTGACCCTGTC |
| 109 | to | 91 | TTTTTTTTTTTTTTTATTCAAACAAAACGAACAC | 25 | to | 7 | TTTTTTTTTTTTTTTCCACTACCTGACCCTGTCC |
| 108 | to | 90 | TTTTTTTTTTTTTTTTTCAAACAAAACGAACACT | 24 | to | 6 | TTTTTTTTTTTTTTTCACTACCTGACCCTGTCCT |
| 107 | to | 89 | TTTTTTTTTTTTTTTTCAAACAAAACGAACACTC | 23 | to | 5 | TTTTTTTTTTTTTTTACTACCTGACCCTGTCCTC |
| 106 | to | 88 | TTTTTTTTTTTTTTTCAAACAAAACGAACACTCG | 22 | to | 4 | TTTTTTTTTTTTTTTCTACCTGACCCTGTCCTCG |
| 105 | to | 87 | TTTTTTTTTTTTTTTAAACAAAACGAACACTCGA | 21 | to | 3 | TTTTTTTTTTTTTTTTACCTGACCCTGTCCTCGA |
| 104 | to | 86 | TTTTTTTTTTTTTTTAACAAAACGAACACTCGAG | 20 | to | 2 | TTTTTTTTTTTTTTTACCTGACCCTGTCCTCGAT |
| 103 | to | 85 | TTTTTTTTTTTTTTTACAAAACGAACACTCGAGA | 19 | to | 1 | TTTTTTTTTTTTTTTCCTGACCCTGTCCTCGATT |
| 102 | to | 84 | TTTTTTTTTTTTTTTCAAAACGAACACTCGAGAT |  |  |  |  |
